# Selective recruitment of beneficial microbes in the rhizosphere of maize affected by microbial inoculants, farming practice, and seasonal variations

**DOI:** 10.1186/s40793-025-00729-y

**Published:** 2025-06-12

**Authors:** Ioannis D. Kampouris, Theresa Kuhl-Nagel, Jan Helge Behr, Loreen Sommermann, Doreen Babin, Davide Francioli, Rita Zrenner, Susanne Kublik, Michael Schloter, Uwe Ludewig, Kornelia Smalla, Günter Neumann, Rita Grosch, Joerg Geistlinger

**Affiliations:** 1 Julius Kühn Institute (JKI) - Federal Research Centre for Cultivated Plants, Institute for Epidemiology and Pathogen Diagnostics, Braunschweig, Germany; 2 Plant-Microbe Systems, Leibniz Institute of Vegetable and Ornamental Crops (IGZ), Großbeeren, Germany; 3 Department of Agriculture, Ecotrophology and Landscape Development, Anhalt University of Applied Sciences, Bernburg, Germany; 4 Department of Nutritional Crop Physiology, Institute of Crop Science, University of Hohenheim, Stuttgart, Germany; 5 Department of Soil Science and Plant Nutrition, Hochschule Geisenheim University, Geisenheim, Germany; 6 Institute for Comparative Microbiome Analysis, Helmholtz Zentrum München – German Research Center for Environmental Health, Neuherberg, Germany; 7 Chair for Environmental Microbiology, Technical University of Munich, München, Germany

**Keywords:** Microbiome, Fungi, Bacteria, Amplicon sequencing, Metagenome sequencing, Rhizosphere competence, Plant stress responses, Iron acquisition

## Abstract

**Background:**

Plant beneficial microorganisms as inoculants can improve crop performance, but factors affecting their impact on plant performance under field conditions remain unclear, thereby limiting their use in farming. Here, we investigated how farming practices (e.g., tillage and N-fertilization intensity) and growing seasons influenced the impact of a beneficial microorganism consortium (BMc: *Trichoderma*, *Bacillus*, and *Pseudomonas* strains) in maize and affected the rhizosphere competence of each BMc strain. In addition, we tested whether the consortium affects the resident rhizosphere microbiome and crop performance. In two growing seasons (2020 and 2021), we assessed how BMc inoculation affects maize growth, nutritional status, gene expression, and rhizosphere microbiome under different farming practices at the flowering stage.

**Results:**

Inoculated strains successfully colonized the maize rhizosphere independently of farming practice. BMc inoculation improved plant growth and iron uptake in 2020, regardless of farming practice. These effects co-occurred with lower precipitation levels in 2020 compared to 2021. BMc inoculation reduced the expression of several stress-related genes in maize in 2020 under drought. An increased iron uptake by the BMc-inoculated plants was observed in 2020 and was associated with the upregulation of the gene *ZmNAS3*, which is linked to iron uptake. Therefore, BMc inoculation mitigated the drought impact on maize. The microbial rhizosphere communities were altered by BMc inoculation in both years, but patterns of responder taxa differed between seasons. Metagenome analysis revealed that more genes (e.g., genes encoding biosurfactants and siderophores) were enriched in the rhizosphere of BMc-inoculated plants in 2020 than in 2021. Moreover, we identified bacterial and fungal taxa positively associated with maize iron uptake. The relative abundance of these iron uptake-associated bacterial and fungal taxa significantly increased due to BMc inoculation in 2020, while they showed overall higher relative abundances in 2021, independently of BMc inoculation. We mapped the sequences of these iron-associated taxa to publicly available genomes and verified the occurrence of various plant beneficial traits in several mapped genomes.

**Conclusions:**

Overall, we show that the growing season determined the effect of BMc inoculation on maize plants by shaping microbiome composition and function in the maize rhizosphere more than farming practice. These findings highlight the importance of the complex interplay between microbial inoculants and the resident rhizosphere microorganisms under abiotic stress conditions.

**Supplementary Information:**

The online version contains supplementary material available at 10.1186/s40793-025-00729-y.

## Background

Agricultural systems are complex environments where crops constantly face multiple biotic and abiotic stress factors that can limit their productivity [1]. To ensure high crop yield, intensified agricultural practices with high inputs of agrochemicals have been applied in the last years; however, these practices are often associated with adverse environmental effects [2]. For instance, agrochemicals can lead to environmental pollution, compromising plant and even human health [3, 4]. Moreover, the diminishment of natural resources and the increasing impact of climate change magnify such problems to a great extent [5].

Harnessing the capabilities of plant-microbiota interactions may contribute to the sustainability of agricultural production systems since plant beneficial microorganisms mediate important key functions in the soil ecosystem, such as nutrient cycling and plant productivity [6]. Specifically, over millions of years, beneficial relationships between plants and microorganisms have been developed (e.g., mycorrhizal fungi, plant growth-promoting rhizobacteria, and endophytes), often based on nutritional and defensive mutualism [7–9]. Consequently, rhizosphere microbiomes play a pivotal role in plant performance, including host fitness, health, and productivity [10], especially under changing environmental conditions [11]. Utilizing plant-associated microorganisms with beneficial effects as inoculants in agriculture has increasingly been recommended as a sustainable management practice to address ongoing economic and ecological challenges [12, 13]. Moreover, the requirement to reduce the input of agrochemicals (e.g., fertilizers, pesticides) by enhancing the productivity of sustainable plant production systems has caused changes in agricultural legislation and policymaking in several countries [14]. Various agricultural policies currently recommend using beneficial microbial inoculants as an environmentally friendly method in sustainable agriculture [15].

Beneficial microorganisms can directly or indirectly support plants. Inoculating beneficial microorganisms, in particular, can directly promote plant growth by improving plant nutrient acquisition under nutritional deficiency and abiotic stress conditions [16–18] or stimulating root growth by modulation of phytohormonal balances through the production of microbial compounds such as indole acetic acid (IAA) or enzymes like 1-aminocyclopropane-1-carboxylate (ACC) deaminase [18–21]. Several inoculants can indirectly enhance tolerance to biotic and/or abiotic stress factors [7, 21], prevent oxidative damage of plant photosystems [22], or produce antimicrobial compounds that inhibit potentially plant pathogenic microorganisms, thereby reducing disease incidence and severity [23]. These effects assist crop adaptation to stress conditions, such as drought [18, 21, 23].

Microbial inoculants must successfully establish in the rhizosphere and maintain their populations over time to interact with plants and benefit plant performance [24, 25]. However, the success of inoculated beneficial microorganisms under field conditions can vary, often showing inconsistent effects on the crops, even under comparable experimental setups at the same location [15, 26]. Various biotic factors (such as plant root exudation patterns or competition with resident rhizosphere microorganisms) and abiotic factors influence the ability of inoculants to colonize the rhizosphere [27]. Farming practices such as tillage and fertilization strongly affect the composition and function of microbiomes in both soil and the rhizosphere, impacting plant performance [28–36]. Since agricultural practices and environmental conditions determine the assembly of soil and rhizosphere microbiomes, they could also influence the fitness and function of inoculated beneficial microorganisms. Our previous results indicated that drought might have affected the outcome of inoculation with a beneficial microorganism consortium (BMc), since inoculation improved the growth of field-grown maize and mitigated the impact of drought on plants under field conditions, with N-fertilization intensity slightly promoting these effects [36].

Because of the impact of farming practice on the assembly of resident rhizosphere microbiomes, we hypothesized that both farming practices and the conditions in the growing season might alter the rhizosphere competence of BMc inoculants and thus maize performance. Thus, in the present study, we aimed to investigate whether farming practice and the conditions of growing seasons impact the effects of BMc inoculation on maize growth, nutrient status, and stress resilience. In addition, we analyzed whether BMc inoculation affected the taxonomic and functional assembly of the resident rhizosphere microbiome in case of successful BMc rhizosphere colonization. To address these objectives, we drench-inoculated maize roots with a BMc in independent field trials at the same location in two consecutive growing seasons in a long-term field experiment that incorporated two contrasting tillage and nitrogen (N) fertilization regimes. At the flowering stage, we measured plant growth, nutrient status, and the expression of genes related to plant nutrient uptake and stress responses. We also determined the rhizosphere establishment of the individual strains of the BMc using cultivation-based methods, and analyzed bacterial and fungal community composition via high-throughput amplicon and shotgun sequencing. In addition, we performed modelling with the amplicon sequencing data and analyzed publicly available genomes that mapped to the amplicons that correlated with the iron shoot concentrations in maize.

## Materials and methods

### Experimental design

The field experiments were established in two growing seasons (2020 and 2021) within a long-term field trial (since 1992) in Bernburg, Germany (51° 82’ N, 11° 70’ E). The soil was characterized as loess chernozem soil over limestone (8% sand, 70% silt, 22% clay; pH 7.0-7.4), as previously described [30]. The ongoing field trial includes plots (1.2 ha) with differently managed soil strips: mouldboard plough (MP, 20–30 cm depth, soil inversion) and cultivator (CT, 12–15 cm depth, soil loosening). These plots are further subdivided into strips with standard N-fertilization intensity (100 kg N ha^− 1^), including the application of pesticides (intensive, Int) and reduced N-fertilization intensity (40 kg N ha^− 1^) without fungicide use (extensive, Ext, Fig. S1). We investigated the effects of a beneficial microorganisms consortium (BMc: *Pseudomonas* sp. RU47 [37], *Bacillus atrophaeus* ABi03 [26], and *Trichoderma harzianum* OMG16 [26]) on plant performance and rhizosphere microbiome in two growing seasons using maize as a model plant (*Zea mays*, cv. Benedictio, KWS Saat SE & Co. KGaA). The strains were selected based on their plant beneficial properties in accordance with previous studies [26]. In total, there were four different combinations of farming practices without and with BMc inoculation, i.e., a total of eight treatments (MP-Ext, MP-Ext + BMc, MP-Int, MP-Int + BMc, CT-Ext, CT-Ext + BMc, CT-Int, CT-Int + BMc, Fig. S1) with four replicates in each growing season. Winter wheat (*Triticum aestivum*) was the pre-crop of maize in both years. Subplots with 33 control plants and 33 BMc-treated plants per replicate were arranged in each investigated growing season. These subplots contained three rows with a distance of 14 cm between the plants and an inter-row distance of 60 cm.

### Preparation of BMc inoculum and application

ABiTEP GmbH (Berlin, Germany) provided a spore suspension of a rifampicin-resistant *Bacillus atrophaeus* ABi03 strain (DSM 32285). The rifampicin-resistant *Pseudomonas* sp. RU47 strain (strain collection of the Julius Kühn Institute, DSM 117411) was cultivated in nutrient broth (Sifin diagnostics GmbH, Berlin, Germany) supplemented with rifampicin (75 µg ml^− 1^; Th. Geyer GmbH & Co. KG, Renningen, Germany) on a rotary shaker (200 rpm) for 24 h at 28 °C. RU47 cells were harvested via centrifugation during the exponential growth stage and suspended in NaCl 0.9% (w/v). The inoculum of *Trichoderma harzianum* OMG16 (strain collection of Anhalt University of Applied Sciences, DSM 32722) was prepared as previously described [38]. Briefly, OMG16 conidia were produced on potato dextrose agar plates (Carl Roth, Karlsruhe, Germany), which were incubated for 20 days at room temperature. Spores were harvested from the plates by adding 5 ml of sterile deionized water. To separate the conidia from the mycelial fragments, the suspension was filtered through a single layer of Miracloth (Merck, Darmstadt, Germany). The conidia density was determined with a hemocytometer and adjusted to the final density. The individual BMc strains were mixed immediately before drenching the plants. BMc inoculation was performed twice manually by applying 50 ml of BMc suspension (10^8^ cells ml^− 1^ for each BMc strain) directly to the stem base of each plant, two and five weeks after emergence (BBCH 12 and BBCH 14). Sterilized tap water (50 ml) was used for control plants.

### Sampling

Samples for determining the rhizosphere competence, nutrient status, gene expression, and microbiome analyses were taken in July 2020 and July 2021 at BBCH 53–63, i.e., 13 weeks of cultivation and four to five weeks after the second inoculation (15/07/2020 and 26/07/2021). For gene expression analysis, a sample (approx. 2 × 2 cm from the middle portion of the lamina) from the youngest fully developed leaves of three maize plants (technical replicates) per biological replicate (*n* = 4) was taken at the flowering stage. The three technical replicates of leaf samples were pooled per biological replicate and immediately immersed in a total of 10 ml RNAlater solution (Thermo Fisher Scientific, Darmstadt, Germany), incubated at 4 °C overnight, and then stored at -80 °C until further processing. The shoot dry mass (SDM) of the same three technical replicate plants was evaluated individually and determined as previously described [35], which preserved the samples for follow-up nutrient analysis. For microbiome analysis, samples from the rhizosphere were taken from the same three plants per replicate and processed as described previously [39].

### Verification of rhizosphere competence

Root samples of the same three technical replicate plants were briefly washed with sterile tap water, pooled (5 g of roots), and processed using a Stomacher 400 Circulator (Seward Ltd., Worthing, UK), as previously described [40]. Briefly, total community DNA was extracted from rhizosphere pellets using the FastPrep-24 bead-beating system and FastDNA Spin Kit for Soil (MP Biomedicals, Santa Ana, CA, USA) following the manufacturer’s protocol. The GeneClean Spin Kit (MP Biomedicals, Santa Ana, CA, USA) was used to purify DNA samples further. The rhizosphere competence of the individual ABi03 and RU47 strains per biological replicate was determined from aliquots of the same rhizosphere pellets of the pooled technical replicates as previously described [26]. Specifically, colony forming units (CFUs) were determined and calculated per gram of root dry mass on Nutrient Agar (Sifin diagnostics, Berlin, Germany) supplemented with 75 µg ml^− 1^ rifampicin and 100 µg ml^− 1^ cycloheximide (Serva Electrophoresis, Heidelberg, Germany). Root-associated soil was obtained by shaking off soil loosely adhering to the roots and used to determine OMG16 with *Trichoderma* selection medium, which was prepared as previously described [39]. The petri dishes with *Trichoderma* selection medium were incubated at 28 °C in the dark for ten days. CFUs were quantified per gram of soil dry mass, which was obtained by drying 5 g of fresh root-associated soil at 110 °C until constant weight. To exclude cross-contamination of the control plots with the BMc strains (due to dispersion from wind or other environmental factors over time), rhizosphere and root-associated soil samples from the control plants were included in the CFU analysis.

### Nutrient analysis

Following SDM estimation, we used the same pooled material and four replicates for determining the nutrient status in maize shoots according to the certified protocols of the Association of German Agricultural Analytic and Research Institutes, VDLUFA, as previously described [39]. Briefly, dry plant material was solubilized by microwave digestion at 210 °C for 25 min. Concentrations of K, P, Mg, S, Ca, Mn, Cu, Fe, and Zn were determined via inductively coupled plasma optical emission spectrometry (ICP-OES, Thermo Fisher Scientific, Dreieich, Germany), whereas total C and N were determined via elemental analysis (Elementary Vario El cube; Langenselbold, Germany).

### RNA extraction and gene expression analysis by RT-qPCR

Pooled leaf samples per replicate were used for gene expression analyses of 26 selected stress-related genes and genes related to nutrient uptake and metabolism. The RNeasy Plant Mini Kit (Qiagen GmbH, Hilden, Germany) was used to extract total RNA from 100 mg of homogenized leaf material, quantified spectrophotometrically (NanoPhotometer NP80; Implen GmbH, Germany), and quality controlled using a 2100 Bioanalyzer and RNA 6000 Nano Kit (Agilent Technologies, USA). Single-stranded cDNA synthesis from 2 µg of total RNA and subsequent qPCR was performed as previously described [34], except that reaction volumes were reduced to 10 µL and a Thermal Cycler CFX96 C1000 Touch was used (Bio-Rad Laboratories GmbH, Feldkirchen, Germany). The genes and their primer pairs are listed in Table S1.

### Analyses of bacterial communities by 16S rRNA gene amplicon sequencing

Library construction and sequencing of the 16S rRNA gene, including positive and negative amplification controls, was carried out by Novogene (Cambridge, UK) on NovaSeq 6000 PE250 (Illumina, San Diego, CA, USA) using the 16S rRNA gene primers Uni341F (5’-CCTAYGGGRBGCASCAG-3’) and Uni806R (5’- GGACTACNNGGGTATCTAAT-3’) [41]. Negative controls obtained from the DNA isolation kit with no soil added indicated very rare occurrence of kit contaminants. The program cutadapt (v3.7) [42] was used to remove primers, barcodes, and adapters. Paired-end reads were processed using the DADA2 pipeline (v1.26.0) [43]. The procedure was performed as recommended by DADA2 developers, including a cutoff based on the size of merged reads to exclude non-specific primer binding. Amplicon sequence variants (ASVs) were classified to the lowest possible taxonomic level by using a Naive Bayesian Classifier [44] trained on the SILVA small subunit of rRNA gene reference taxonomy database (v138.1) [45]. Sequencing depth sufficiently covered diversity in each sample (Fig. S2). One sample from the control of MP-Int was excluded from further analysis because it was poorly sequenced (< 20,000 reads). Additionally, sequences with less than five reads and sequences identified as chloroplasts or mitochondria were removed based on their taxonomic classification from SILVA. Our analysis produced 2,433,907 merged reads from 7,691,757 raw reads with 38,029.8 ± 7,068.67 reads per sample (Fig. S2) and 13,252 ASVs in total. To minimize the effects of uneven sequencing/sampling depth and compositionality on ASV abundance [46], we performed repeated rarefactions. Specifically, we rarefied to 20,000 reads via a repeated rarefaction process (1,000 times), and the average read abundances were calculated. No archaeal sequence was present in the dataset after the preliminary processing steps due to their removal at the size cutoff step.

### Analyses of fungal communities by ITS2 amplicon sequencing

The Internal Transcribed Spacer 2 (ITS2, ITS86F/ITS4 primer pair) was used to analyze fungal communities in the rhizosphere as previously described [26]. Briefly, three PCRs per sample with 10 ng soil DNA and bovine serum albumin (BSA, 0.5 mg m^− 1^) were carried out at three annealing temperatures (56 °C ± 2 °C) using 25 cycles and a 20 µl volume with Q5^®^ High-Fidelity 2x Master Mix (New England Biolabs, Frankfurt, Germany). Negative controls obtained from the DNA isolation kit with no soil added were run for each barcode primer while preparing the sequencing pools. No amplification products were detectable with the Qubit fluorometer (Thermo Fisher Scientific, Schwerte, Germany). High-throughput sequencing was carried out on the Illumina^®^ MiSeq^®^ platform (Illumina, San Diego, CA, USA) in paired-end mode (2 × 300 bp) as previously described [35]. Taxonomic assignment based on a database-dependent strategy [47] using the GALAXY bioinformatics platform and UNITE v9.0 database [48, 49] was performed as previously described [50]. After removing singletons, 6,455,222 reads were obtained, resulting in 100,863 ± 16,553 reads per sample (Fig. S2) and 2,413 ASVs. ASV abundance was rarefied to 48,690 via a repeated rarefaction process (1000 times), and the average read abundances were calculated.

### Metagenome sequencing

A multiplexed metagenomic library was prepared from rhizosphere DNA using NEBNext Ultra II FS DNA Library Prep Kit^®^ (New England Biolabs, Frankfurt, Germany) as previously described [35]. Sequencing was performed on the NextSeq 550 sequencer (Illumina, San Diego, CA, USA) using the NextSeq 500/550 High Output Kit v2.5 (300 cycles). Raw reads were processed as previously described [35]. Coverage of the metagenomic dataset was estimated using Nonpareil (GALAXY Version 3.1.1.0) [35] in alignment mode, and results indicated sufficient coverage for read-based analysis (Fig. S3). After pre-processing, 344,628,088 sequences were obtained for 2020, representing 91.3% of the raw reads (Table S2). For 2021, pre-processing yielded 321,676,174 sequences, accounting for 89.3% of the raw reads (Table S2). Taxonomic and functional annotation was performed using the GALAXY bioinformatics platform as previously described [35]. Sequences annotated as bacterial were extracted for further analysis. Only a minor fraction of sequences was annotated as fungi (2020: 0.04%, 2021: 0.06%) and archaea (2020: 0.8%, 2021: 0.8%); thus, fungal and archaeal sequences were excluded from further analysis. To analyze potential plant beneficial bacterial functions, a customized database was applied, as previously [35]. The annotation of sequences based on the COG (Clusters of Orthologous Genes) database identified 4,333 genes for 2020 and 4,340 genes for 2021. Using the customized database, 353 genes were identified in 2020, corresponding to 1,540,927 sequences (1.7%), while 351 genes were detected in 2021, corresponding to 1,431,559 sequences (1.5%). For the detection of the inoculated BMc strains in the rhizosphere, sequences taxonomically annotated as *T. harzianum*, *B. atrophaeus*, and *Pseudomonas* sp. were extracted, and their differential abundance between inoculated and control rhizosphere samples was further analyzed. To confirm whether sequences corresponded to the inoculated microorganisms, sequences were compared with the respective genomes of each BMc strain using BLAST [51] with 100% sequence identity and 100% sequence alignment.

## Additional data analysis and statistics

### Plant and nutrient data

Statistical analysis of plant growth and nutrient content was performed using R (v.4.2.2) [52]. Main and interaction effects between different long-term farming practices and BMc inoculation and year were analyzed by linear models (LM) for SDM, shoot and soil nutrients, and BMc rhizosphere competence. The normality of residuals was inspected either using the Shapiro’s test or visually when the Shapiro’s test indicated slight deviances from normality of residuals. Pairwise comparisons were conducted using Dunn’s test with Benjamini-Hochberg correction (package “dunn.test”, v1.3.6). Data were visualized using “ggplot2” (v.3.4.1) [53]. Furthermore, to evaluate which nutrients were limiting factors for plant growth, we generated a structural equation model (SEM) based on partial least squares, with nutrient shoot concentrations and the nutrient uptake and SDM using the package “lavaan” (v0.6.17) [54]. All comparisons with *p*-values of 0.05 or lower were considered as statistically significant (*α* = 0.05). In addition, we considered the proposed nutrient limitation status for maize plants [55]. Our experiment followed a multi-factorial procedure of three binary factors and four replicates per group, which resulted in 32 samples per year and 64 samples in total. Consequently, comparing a single factor (e.g., BMc inoculation) per year resulted in 16 replicates per treatment. Applying models that utilized all the data from one year resulted in 32 samples, and applying all the data from the two years resulted in 64 samples (Fig. S1).

### Analysis of gene expression

The selected maize genes for biotic and abiotic stress responses, nutrient uptake, and metabolism were chosen for expression analyses based on previous studies with other plant species [34, 35] and are listed in Table S1. For calculating gene expression levels, the ΔΔCq method, as previously described [56], was employed for relative transcript quantification. The averages of three technical replicates were normalized to the average of the expression level of the endogenous controls. The relative transcript levels (ΔCq = housekeeping genes Cq - target gene Cq) were calculated from BMc-treated and control plants, and the differential abundance was computed (log_2_FC = ΔCq BMc – ΔCq Ctrl). For each condition, the mean value was calculated for each of the four biological replicates (*n* = 4). Significant differences between BMc-inoculated and control plants were calculated using the t-test (*α* = 0.05).

### Analysis of amplicon and metagenome sequencing data

Microbiome analysis based on amplicon sequencing was performed using R (v.4.2.2) [52], the “tidyverse” set of packages (v.1.3.1) [53], and vegan (v.2.6.1) [56]. Samples were analyzed in rarefied abundance for bacterial and fungal communities and presented in the percentage of reads. The Bray-Curtis distance of rarefied data was used for estimating the β-diversity. PERMANOVA tests (10,000 permutations) were applied to evaluate how BMc inoculation and farming practices affected bacterial or fungal β-diversity between the two growing seasons. To investigate the effect of BMc inoculation on bacterial or fungal ASVs, we used logistic regression (BMc vs. Control) with Benjamini-Hochberg correction, considering all possible combinations of different farming practices and growing seasons.

Metagenomic data were analyzed based on the relative abundance of reads by dividing the number of classified reads by the total reads and applying a log_10_ transformation. PERMANOVA analysis (Bray-Curtis dissimilarity, 10,000 permutations) was performed to detect the effects of BMc inoculation, different farming practices, and their interactions. Dissimilarities between samples were visualized with NMDS using the Bray-Curtis distance matrix. Differential analysis of the functional profile of metagenome bacterial genes was performed with the edgeR algorithm (v3.40.2) [57], with a minimum prevalence threshold of one read per three samples.

### Association of bacterial and fungal communities with iron shoot concentrations

Since we observed significant differences in iron concentrations in maize shoots between the growing seasons, we aimed to associate bacterial and fungal taxa with these concentrations. Therefore, we fitted LMs using log_10_ transformed relative abundance with pseudocount addition (+ 0.1) and Benjamini-Hochberg correction. All ASVs with positive β-coefficients and *p* < 0.05 were considered iron-associated. We also estimated the prediction potential of iron-associated ASVs for iron uptake via random forest regression with the package “randomForest” (v4.7-1.1) [58]. To further verify their potential plant beneficial traits, we selected ASVs from the family with the highest number of bacterial iron-associated ASVs as an example. Since short-read shotgun sequencing yielded too low coverage to obtain fully assembled genomes, we mapped iron-associated ASVs to the global taxonomy database (GTDB, release 214) [59] using usearch local alignment (v11) [60] with minimum alignment of 400 bp, minimum percentage identity of 97%, and e-value < 10^− 5^. We annotated the identified genomes via PROKKA (v1.14.5) [61] to predict ACC deaminase and antiSMASH (v. 7.1.0) [62] for siderophore-associated genes, respectively. Furthermore, the effect of BMc inoculation and growing season on the total relative abundance of iron-associated ASVs was estimated via the Wilcoxon rank-sum test (*α* = 0.05).

## Results

### Climatic conditions influenced the plant growth promotion effect of BMc inoculation

**Fig. 1:**
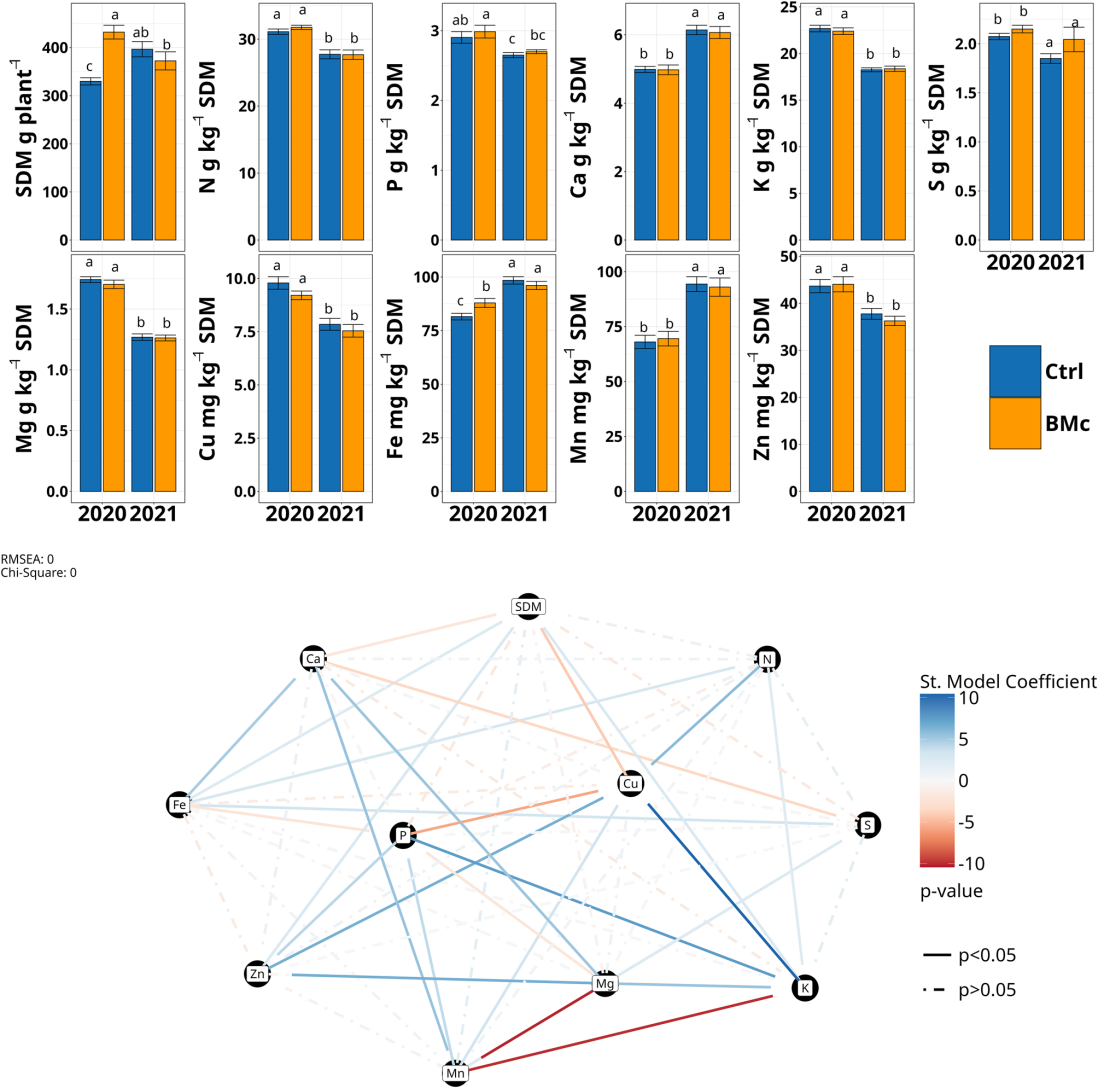
(**A**) Shoot dry mass (SDM) and concentrations of macro- and micronutrients (g or mg) kg^− 1^ SDM of control (Ctrl) and BMc inoculated plants in 2020 and 2021. Different letters mark significant differences between treatments with pairwise Wilcoxon rank-sum test (*p <* 0.05, Benjamini-Hochberg correction, *n* = 16). (**B**) Structural equation model of macro- and micronutrient concentrations in SDM (mg or g kg^− 1^ SDM) and SDM (g plant^− 1^) based on partial least squares. The model shows significant relationships with solid lines (*p* < 0.05). Dashed lines show non-significant relationships (*p >* 0.05). The standardized (St.) model coefficient indicates a positive (blue) or negative (red) correlation (*n* = 64)

During the BMc inoculation experiments, we encountered different weather conditions between the two growing seasons. Spring precipitation (March-May) was below average in 2020 (average spring precipitation 1981–2010 = 42.6 mm, total spring precipitation 2020 = 18.3 mm, Fig. S4). In contrast, total spring precipitation was higher in 2021 than in 2020 (27.6 mm, Fig. S4). We estimated the shoot dry mass (SDM) as a proxy for the performance of maize in the two consecutive years. For single effects, BMc inoculation and N-fertilization intensity explained SDM variance by 7.2% and 4.7%, respectively (LM, *p* < 0.05, *n* = 64, Table S3). Nevertheless, the SDM variance was mainly explained by BMc inoculation and growing season, with their interaction explaining 21.93% of SDM variance (LM, *p* = 0.00002, *n* = 64, Table S3). Specifically, SDM significantly increased due to BMc inoculation from 329.8 ± 29.7 to 432.0 ± 57.0 g plant^− 1^ in 2020 (Wilcoxon rank-sum test, *p* < 0.001, *n* = 16). In contrast, SDM did not significantly differ between BMc and control plants in 2021 (Control: 396.5 ± 63.2, BMc: 372.3 ± 75.3 g plant^− 1^, Wilcoxon rank-sum test, *p* > 0.05, *n* = 16; Fig. 1A). Meanwhile, tillage contributed to the outcome of BMc inoculation since their interaction explained 11.45% of variance in SDM (LM, *p* = 0.0012, *n* = 64, Table S3). Notably, control plants had significantly higher SDM in 2021 than in 2020 (Wilcoxon rank-sum test, *p* < 0.05, *n* = 16; Fig. 1A). Thus, BMc inoculation promoted plant growth mainly in 2020.

We analyzed the nutrient concentrations in maize shoots to link the effect of BMc inoculation with potential influence on nutrient uptake (Fig. 1). Out of all the nutrients, BMc inoculation affected iron concentrations only in 2020 (LM, *p* = 0.008, Table S4) but not the rest of the nutrient concentrations. Iron concentration increased from 81.5 ± 8.4 to 87.9 ± 6.2 mg kg^− 1^ SDM in the year 2020 (Wilcoxon rank-sum test, *p* < 0.05, *n* = 16; Fig. 1A). However, BMc inoculation did not significantly affect iron concentration in 2021 (Control: 96.1 ± 6.9 mg kg^− 1^ SDM, BMc: 98.4 ± 7.6 mg kg^− 1^ SDM, LM, *p* = 0.298, Table S5). Moreover, all plants had significantly higher iron concentrations in 2021 than in 2020 (Wilcoxon rank-sum test, *p* < 0.05, *n* = 16; Fig. 1A). A higher manganese concentration was also observed in 2021 compared to 2020, but there were no differences between the treatments. In contrast, the concentrations, especially of potassium, zinc, and copper, were significantly lower in shoot samples of 2021 than in 2020 (Wilcoxon rank-sum test, *p* < 0.05, *n* = 16), but not due to BMc inoculation (Wilcoxon rank-sum test, *p* > 0.05, *n* = 16, Fig. 1A).

To identify which nutrients acted as limiting factors for maize growth, we generated a structural equation model (SEM, Fig. 1B), which indicated a significant positive correlation of SDM with iron, zinc, and potassium concentrations in maize shoots (*p* < 0.05, Fig. 1B, Table S6). The iron correlation with SDM was also present when we performed SEM with total nutrient content per plant (*p* < 0.05, *n* = 64, Fig. S5). This suggests that BMc inoculation strongly affected iron uptake in 2020, which was among the three most important limiting factors for plant growth, along with zinc and potassium (Table S6).

### BMc inoculation downregulated the expression of stress-related genes under drought in 2020

**Fig. 2:**
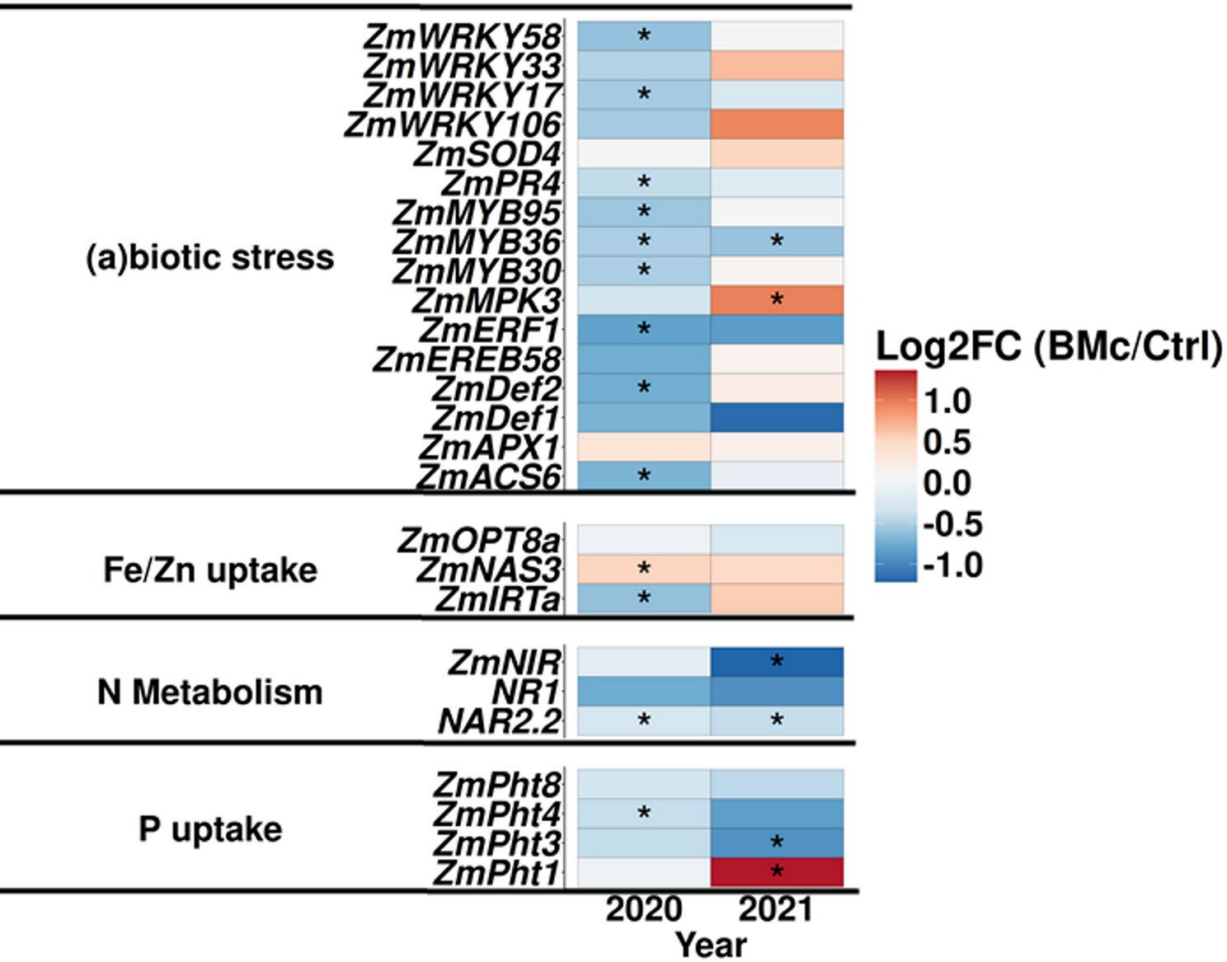
Plant gene expression differences of selected genes in maize (cv. Benedictio) treated with a beneficial microorganism consortium (BMc) and untreated control plants (Ctrl). Maize plants were cultivated in a long-term field experiment (Bernburg, Germany) in the growing seasons of 2020 and 2021. The relative transcript levels (ΔCq = housekeeping genes Cq- target gene Cq) were calculated from BMc-treated and Ctrl plants, and the differential abundance was computed (ΔΔCq = ΔCq BMc – ΔCq Ctrl). Significant differences between BMc-treated and Ctrl plants were verified with Student’s t-test and labeled with asterisks (*p < 0.05, *n* = 16). Log2FC = log_2_ fold change of relative gene expression (BMc ΔCq -Control ΔCq). Detailed gene assignments can be found in Table S1

To further estimate the impact of BMc inoculation on maize plants in the two growing seasons, we analyzed the expression levels of 26 maize genes in leaves. These genes are related to abiotic/biotic plant stress responses, nutrient uptake, and N-metabolism. Notably, BMc inoculation significantly downregulated the expression levels of nine stress-related genes in 2020 (log_2_ fold-change < -0.51, t-test, *p* < 0.05, *n* = 16, Fig. 2). Yet, BMc inoculation did not affect all these genes in 2021, except the gene *ZmMYB36* (t-test, *p* > 0.05, *n* = 16, Fig. 2). Consequently, BMc inoculation increased plant stress resilience in 2020 (Fig. 2) when plants were exposed to approximately 20% lower precipitation in spring (Fig. S4). Furthermore, BMc inoculation upregulated the expression of the gene *ZmNAS3* in both years but significantly only in 2020 (log_2_ fold-change = 0.53, t-test, *p =* 0.012, *n* = 16; 2021: log_2_ fold-change = 0.50, t-test, *p* = 0.08, *n* = 16, Fig. 2). This gene is directly connected with nicotianamine synthesis, which is a metal-chelating molecule (Table S1), corroborating our shoot nutrient results for 2020 (Fig. 1A). In contrast, BMc inoculated plants showed significantly downregulated expression levels of the *ZmIRTa* gene (encoding an iron transporter protein) only in 2020 (log_2_ fold-change = -0.61, t-test, *p* < 0.05, *n* = 16, Fig. 2). In addition, BMc inoculation upregulated the expression of *ZmNAR2.2* gene in 2020 and 2021 (log_2_ fold-change > 0.31, t-test, *p* < 0.05, *n* = 16). Moreover, a few genes associated with phosphorus (P) uptake (*ZmPht1* and *ZmPht3*) and N-metabolism (*ZmNIR* and *ZmNAR2.2*) were affected only in 2021 (*ZmPht1* log_2_ fold-change = 1.36, *ZmPht3* log_2_ fold-change = -0.93, *ZmNIR* log_2_ fold-change = -1.22, t-test, *p* < 0.05, *n* = 16, Fig. 2). In conclusion, the gene expression data indicate that BMc inoculation impact on iron uptake was linked to the downregulation of stress-associated genes.

### BMc strains established in the rhizosphere independently of farming practice and growing season

**Fig. 3:**
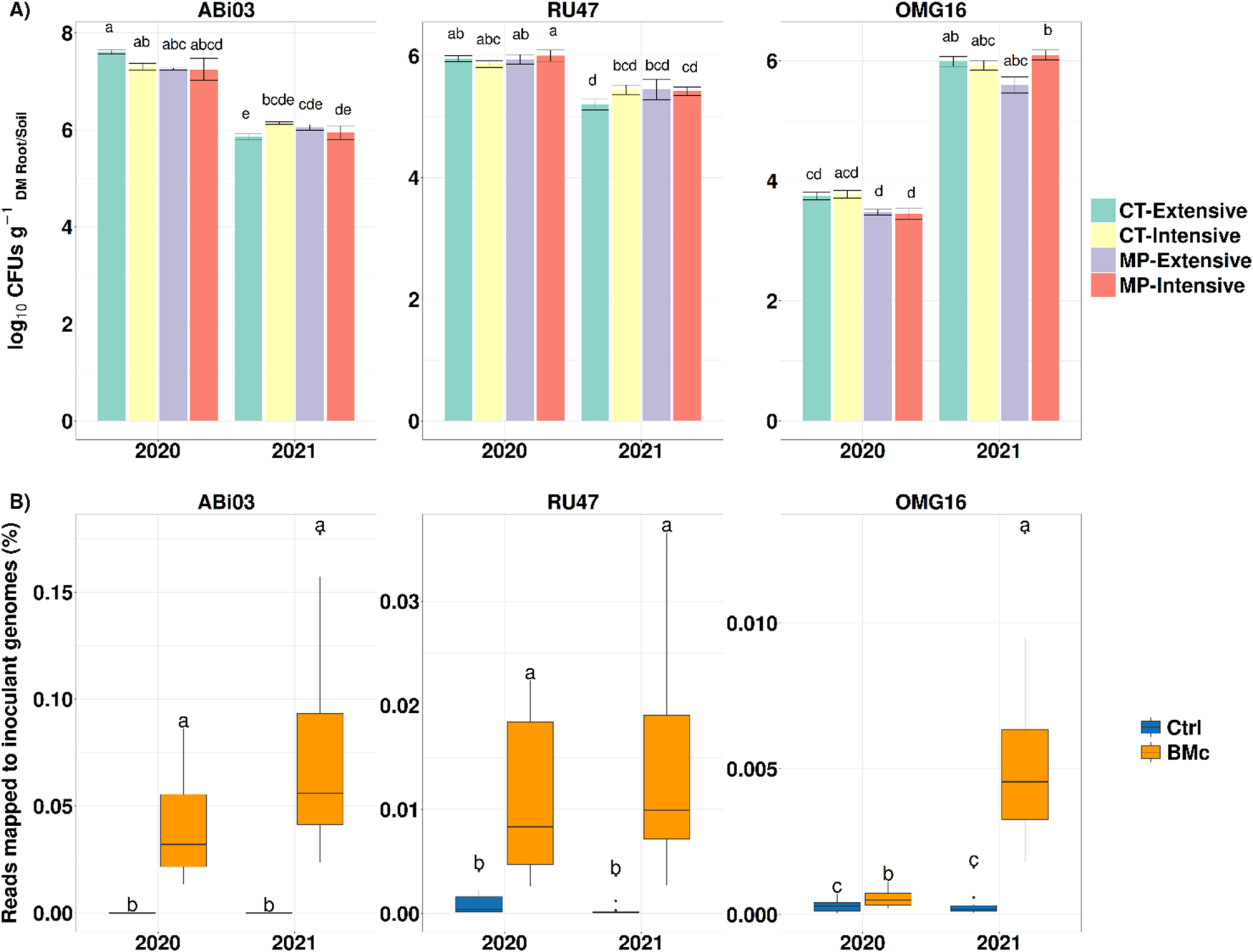
(**A**) Colony forming unit (CFU) counts for each microbial strain based on gram of root dry mass (*Pseudomonas* sp. RU47 and *Bacillus atrophaeus* ABi03) and gram of dry root-associated soil (*Trichoderma harzianum* OMG16) of the applied beneficial microorganism consortium (BMc). Statistical differences were tested with pairwise Wilcoxon rank-sum tests (Benjamini-Hochberg correction, *n* = 16). Different letters indicate significant differences (*p* < 0.05) for each strain in the different farming practices and growing seasons. (**B**) Relative abundance of reads mapped to the inoculant genomes in the maize rhizosphere. Different letters indicate significant differences (*p* < 0.05) for each inoculant genome in response to inoculation and growing season tested with pairwise Wilcoxon rank-sum test (Benjamini-Hochberg correction, *n* = 16). DM: dry mass, CT: cultivator tillage, MP: mouldboard plough tillage, Ctrl: untreated control plants

To elucidate whether the interaction effect of growing season and BMc inoculation on SDM (Fig. 1) can be explained due to differences in the rhizosphere competence of the BMc strains between the two years, we performed cultivation-based and cultivation-independent methods. All strains colonized the maize rhizosphere independently of farming practice in both years, but with differences in their densities depending on the year (LM, *p* < 0.05, *n* = 4, Fig. 3A). Neither tillage nor N-fertilization intensity affected the densities of the bacterial strains (ABi03, RU47) in the rhizosphere and the root-associated soil (RAS) densities of OMG16 (Wilcoxon rank-sum test, *p* > 0.05, *n* = 16, Fig. 3A). Both bacterial strains of the consortium colonized the rhizosphere of inoculated plants and remained present in both years with CFU counts ranging for ABi03 from 6.94 to 7.91 log_10_ CFU g^− 1^ root dry mass (RDM) in 2020, from 5.48 to 6.25 log_10_ CFU g^− 1^ RDM in 2021, and for RU47 from 5.74 to 6.14 in 2020 and from 4.95 to 5.94 log_10_ CFU g^− 1^ RDM in 2021. A higher density of the bacterial strains in the rhizosphere was revealed in 2020 compared to 2021 (LM, *p* = 0.001, *n* = 16, Fig. 3A). For OMG16 the range was from log_10_ 3.20 to 3.90 CFU g ^− 1^ dry RAS in 2020 and from log_10_ 5.73 to 6.31 CFU g^− 1^ dry RAS in 2021 (Fig. 3A). No colonies of the bacterial BMc strains were found in the rhizosphere samples of the control plants and a much lower number of *Trichoderma* isolates in the RAS of the controls. In addition, the colonization of the inoculated strains and the absence of cross-contamination were further verified by testing their abundance in the metagenome sequencing data (Fig. 3B). Differential abundance of reads assigned to *B. atrophaeus*, *Pseudomonas* sp., and *T. harzianum* indicated a significantly higher relative abundance of all strains in inoculated compared to control plants by at least one order of magnitude in both years, with similar relative abundances in both years (Wilcoxon rank-sum test, *p* < 0.05, *n* = 16, Fig. 3B). Most of these reads mapped to ABi03, RU47, and OMG16 genomes (Table S7).

### BMc inoculation induced β-diversity shifts in resident microbiomes in both years

**Fig. 4:**
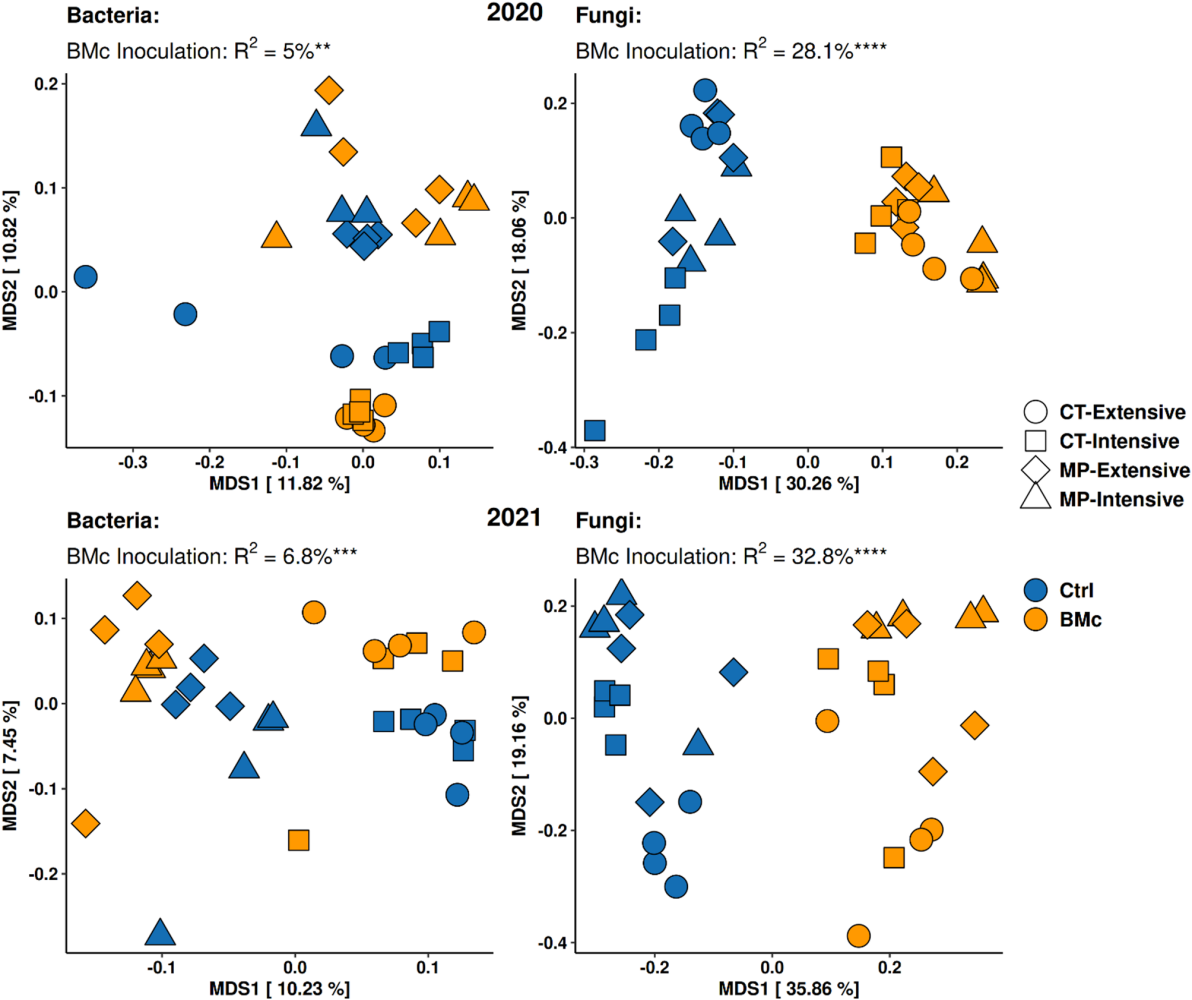
Bacterial and fungal community compositions based on Bray-Curtis distances (MDS: multi-dimensional scaling). Statistical differences between the rhizosphere communities from plants inoculated with a BMc (beneficial microorganism consortium) and control (Ctrl) plants were tested with PERMANOVA (*p < 0.05, **p < 0.01, ***p < 0.001 and ****p < 0.0001, *n* = 32). MP: Mouldboard plough tillage, CT: Cultivator tillage

To elucidate whether the compositional changes in rhizosphere bacterial and fungal communities were associated with the observed plant growth effect due to BMc inoculation, we performed amplicon sequencing of the bacterial 16S rRNA gene and the fungal ITS2 region. Most of the explained variance in β-diversity of bacterial and fungal rhizosphere communities was driven by the differences in growing seasons (PERMANOVA, 16S: R^2^ = 20.9%, ITS: R^2^ = 19.4%, *p* < 0.05, *n* = 32, Table S8, S9). To further investigate how the differences between the two years influenced inoculation and the effects on bacterial and fungal β-diversity, we separated the analysis between the two growing seasons. Tillage practice strongly shaped the assembly of bacterial communities in both seasons (PERMANOVA, R^2^ = 9.7–10.0%, *p* < 0.001, *n* = 16), followed by BMc inoculation (PERMANOVA, R^2^ = 5.0-6.8%, *p* < 0.01, *n* = 16, Fig. 4, Table S10). BMc inoculation and tillage also showed an interaction effect in both growing seasons (PERMANOVA, R^2^ = 4.5–4.9%, *p* < 0.01, *n* = 16). The effect of N-fertilization intensity was lower in both years (PERMANOVA, R^2^ = 3.5–3.9%, *p* < 0.05, *n* = 16). In addition, a significant tripartite interaction was observed in both growing seasons (PERMANOVA, R^2^ = 3.7–4.6%, *p* < 0.05, *n* = 16, Fig. 4, Table S10).

In fungal communities, BMc inoculation caused β-diversity shifts in 2020 (PERMANOVA, R^2^ = 28.1%, *p* < 0.001, *n* = 16) and 2021 (PERMANOVA, R^2^ = 32.8%, *p* < 0.001, *n* = 16, Fig. 4, Table S11). Also, tillage influenced fungal community composition significantly in both growing seasons (PERMANOVA, R^2^ = 5.9–7.1%, *p* < 0.05, *n* = 16). Nevertheless, the interactions between the factors were not as pronounced as in bacterial communities. For example, BMc inoculation significantly interacted with N-fertilization intensity and tillage practice only in 2020 (PERMANOVA, R^2^ = 5.9%, *p* < 0.05, *n* = 16) but not in 2021 (PERMANOVA, R^2^ = 1.4%, *p* > 0.05, *n* = 16, Fig. 4, Table S11). In conclusion, BMc inoculation, as a single or interaction factor with farming practice, generally affected bacterial and fungal β-diversity in both years, with similar effect sizes of single factors and interaction effects between the two years. Thus, the interaction effect of BMc inoculation and growing season on SDM (Table S3) can be explained by the observed microbial β-diversity shifts in the rhizosphere in 2020 but not in 2021 (Fig. 4).

### BMc inoculation induced different taxonomic patterns in bacterial and fungal communities in 2020 and 2021

**Fig. 5:**
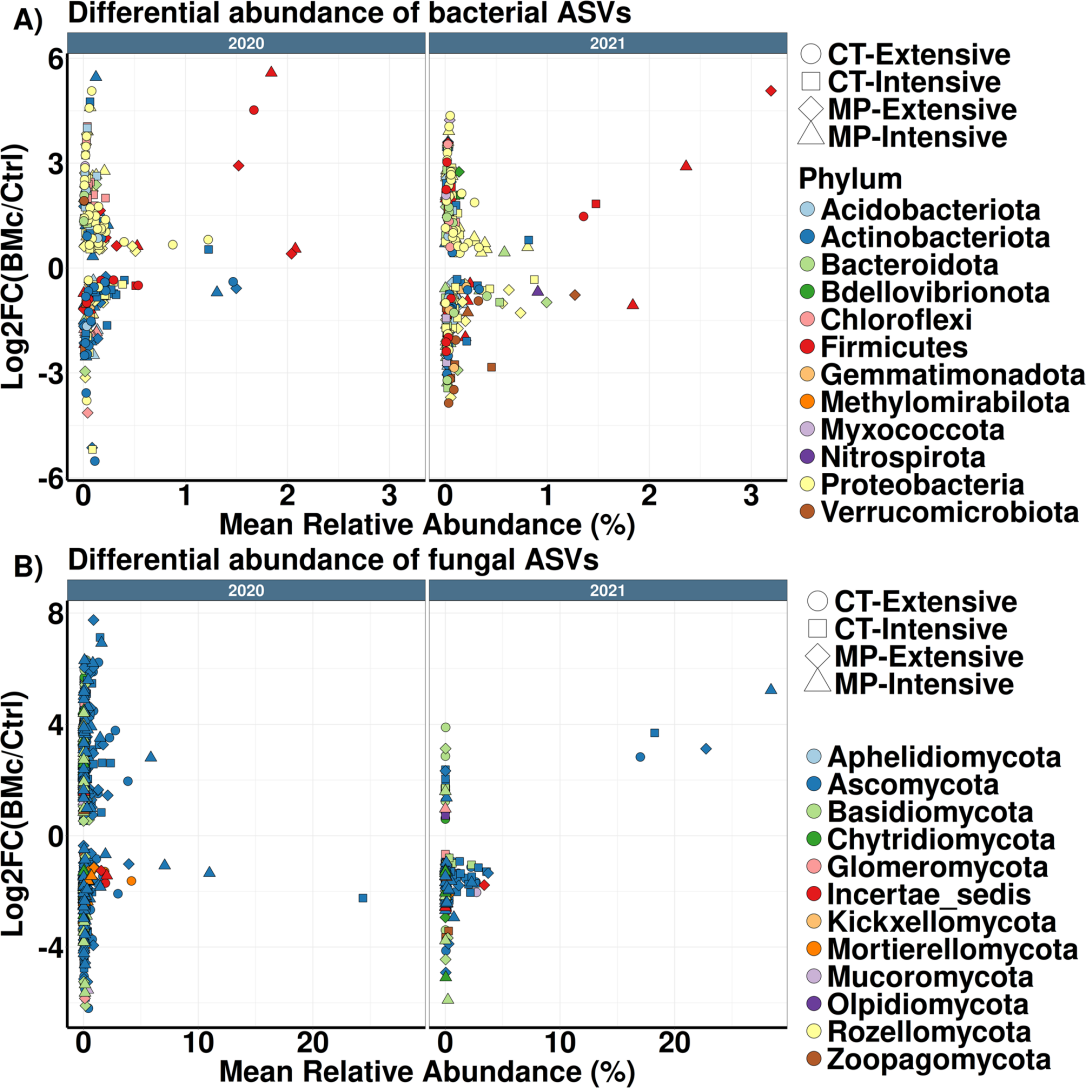
Differentially abundant ASVs (amplicon sequence variants) due to inoculation with a BMc (beneficial microorganism consortium) in the rhizosphere of maize (cv. Benedictio) based on differential abundance tests (BMc vs. Ctrl; control) of bacterial (**A**) and fungal (**B**) ASVs (logistic regression, *p* < 0.05, Benjamini-Hochberg correction). Color depicts the taxonomy of bacterial and fungal ASVs at the phylum level. MP: Mouldboard plough tillage, CT: Cultivator tillage

We analyzed bacterial and fungal community composition for both years to determine whether specific compositional shifts due to BMc inoculation or growing conditions were linked to plant growth. Across all farming practices, BMc inoculation enriched the relative abundance of 149 bacterial ASVs in 2020 and 143 ASVs in 2021, while it reduced the relative abundance of 128 ASVs in 2020 and 142 ASVs in 2021 (logistic regression, *p* < 0.05, *n* = 4, Fig. 5A, Table S12). Despite this, we observed that BMc inoculation had a stronger impact on the taxonomic composition of differentially abundant ASVs in 2020 than in 2021. For instance, 61 ASVs classified as *Actinobacteriota* showed a significant decrease in abundance in 2020 (logistic regression, *p* < 0.05, *n* = 4). In contrast, only 21 ASVs classified as *Actinobacteriota* decreased in 2021 (logistic regression, *p* < 0.05, *n* = 4, Fig. 5A, Table S12). BMc inoculation promoted the increase of 399 fungal ASVs in 2020, whereas only 26 ASVs increased in 2021 (logistic regression, *p* < 0.05, *n* = 16, Fig. 5B, Table S13). However, these 26 fungal ASVs in 2021 showed a high dominance in the rhizosphere, surpassing relative abundances of 1% (Fig. 5B). Despite the differing numbers of responder ASVs, these shifts seemed to be restricted to specific phyla in both years (*Ascomycota*,* Basidiomycota*, and *Kickxellomycota*, Fig. 5B). In addition, the relative abundance of 310 and 167 fungal ASVs decreased due to BMc inoculation in 2020 and 2021, respectively (logistic regression, *p* < 0.05, *n* = 16, Fig. 5B, Table S13).

### BMc inoculation effect on the relative abundance of plant beneficial bacterial genes was stronger in 2020 than in 2021

**Fig. 6:**
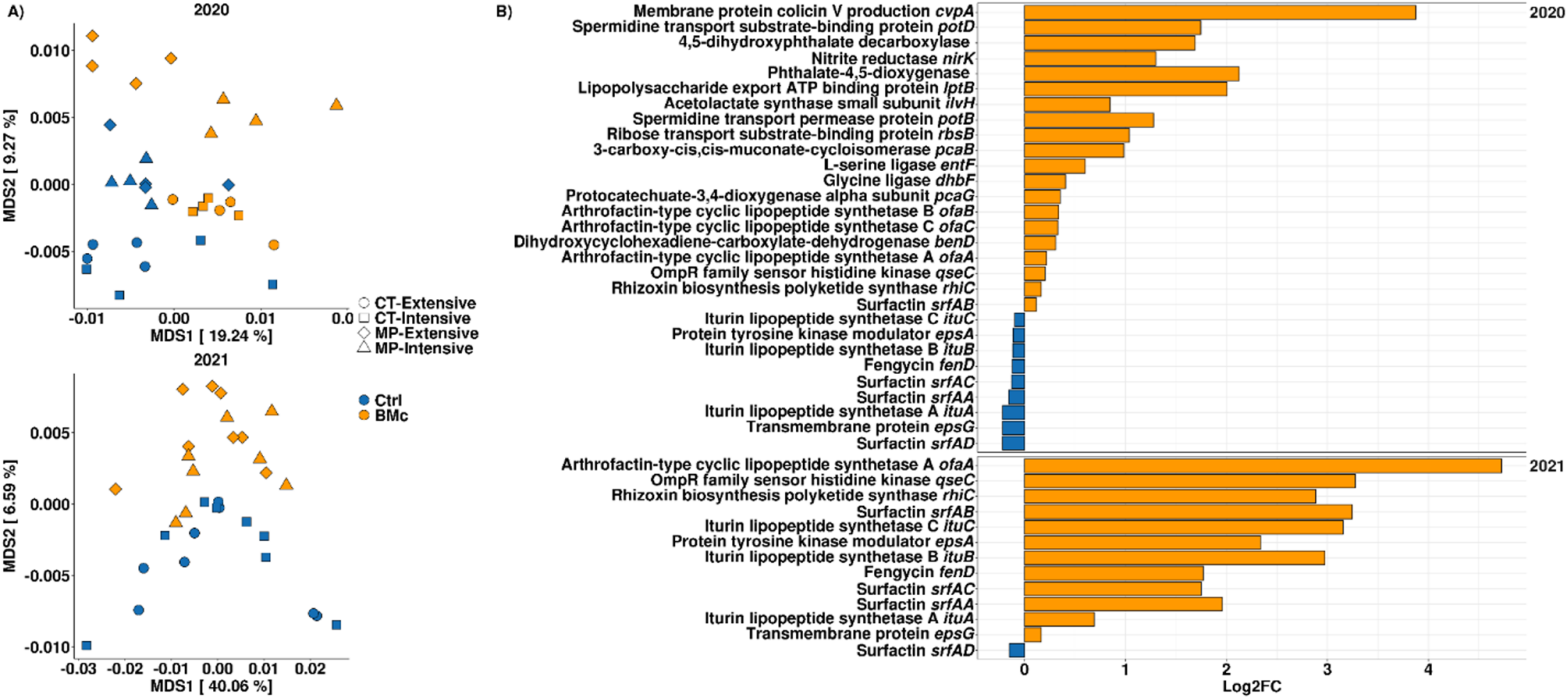
(**A**) Composition of bacterial gene functions in the rhizosphere bacterial community of maize (cv. Benedictio) affected by inoculation with a beneficial microorganism consortium (BMc) compared to untreated control (Ctrl) plants in the growing seasons 2020 and 2021 based on Bray-Curtis dissimilarity (MDS: multi-dimensional scaling). (**B**) Differentially abundant (log_2_ fold change BMc/Ctrl, Log_2_FC) plant beneficial functions (customized database) in the rhizosphere of maize between BMc inoculated and Ctrl plants in growing seasons 2020 and 2021, based on edgeR and Benjamini-Hochberg correction (*p* < 0.05, *n* = 16). The log_2_ fold change of differential gene abundance was derived via functional annotation of rhizosphere metagenomes using a customized COG database for plant beneficial functions

To investigate whether changes in the functional potential of the rhizosphere microbiomes were linked with plant growth, we performed metagenome sequencing of rhizosphere DNA and mapped the sequences to a customized database for plant beneficial genes. BMc inoculation and tillage practice significantly shaped the functional profile in the rhizosphere of maize in both years (PERMANOVA, BMc inoculation: R^2^ = 9.0-10.4%, Tillage: R^2^ = 6.4–7.3%, *p* < 0.001, *n* = 16, Fig. 6A, Table S14). In contrast, an effect of N-fertilization intensity was only observed in 2020 (PERMANOVA, *p* < 0.05, *n* = 16, Fig. 6A, Table S14). A higher portion of bacterial genes responded to BMc inoculation in 2020 (29 genes, edgeR, *p* < 0.05, Benjamini-Hochberg correction, *n* = 16, Fig. 6B) than in 2021 (13 genes, edgeR, *p* < 0.05, Benjamini-Hochberg correction, *n* = 16, Fig. 6B). Moreover, 11 out of these genes associated with bacterial metabolites [e.g. iturin (*ituA*, *ituB*, *ituC*), fengycin (*fenD*), surfactin (*srfAA*, *srfAB*, *srfAC*, *srfAD*)] and siderophore production (e.g. *dhbF*, *entB*, *entF*) and showed higher relative abundances in BMc inoculated plants in both years (Fig. 6B). However, these genes can be found in the *B. atrophaeus* ABi03 genome; thus, reflect the differential abundance of ABi03 in both years. In conclusion, BMc inoculation significantly affected the relative abundance of plant beneficial genes, with a stronger enrichment in 2020 than in 2021.

### Correlation of bacterial and fungal ASVs with iron concentrations in maize shoots

**Fig. 7:**
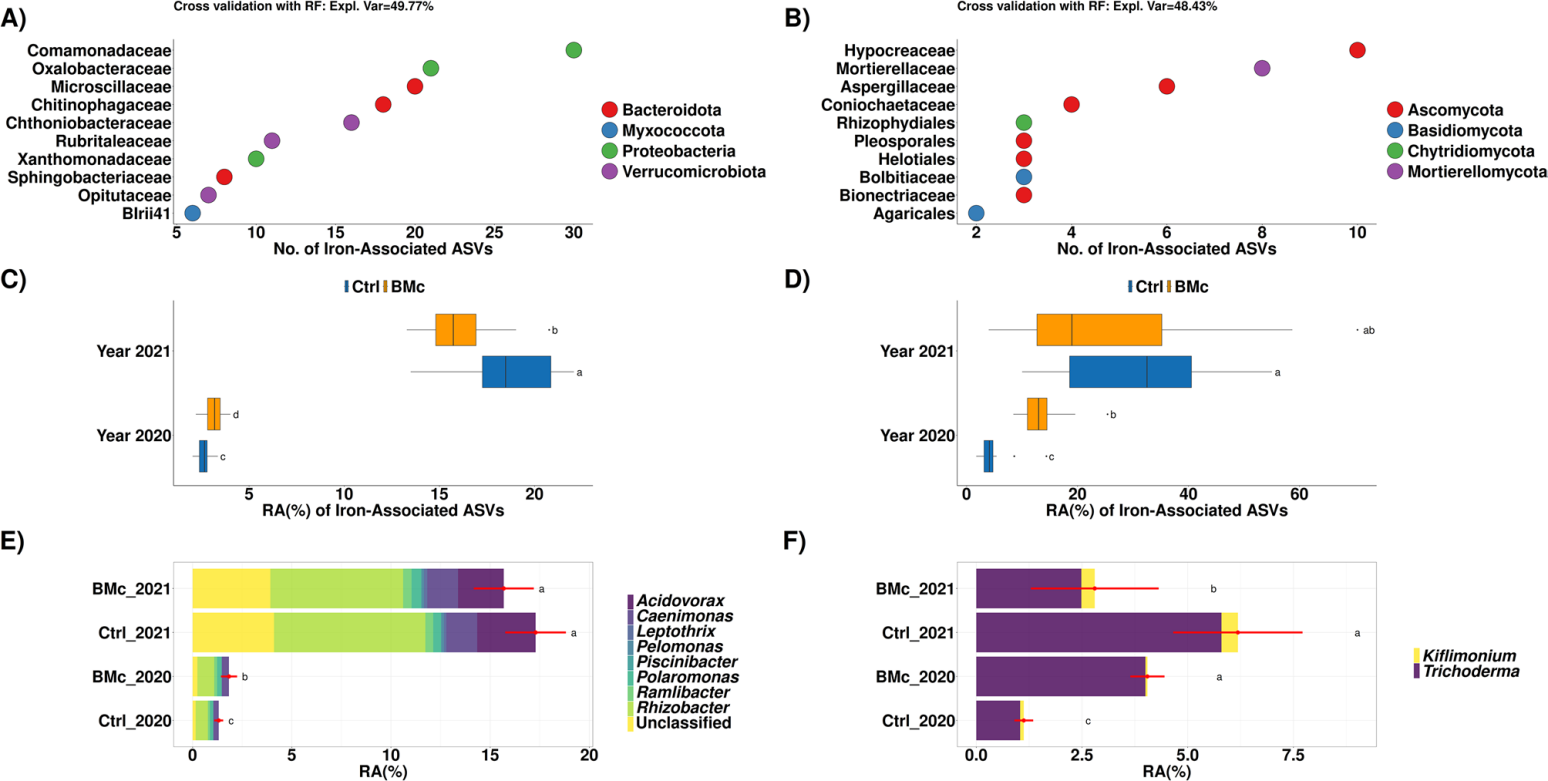
Iron-associated bacterial (**A**) and fungal (**B**) ASVs (amplicon sequence variants) in the rhizosphere of maize (cv. Benedictio) in the year 2020 based on linear regression (*p* < 0.05, *β*-coefficient > 0.005, *n* = 64, Benjamini-Hochberg correction). The capability of the iron-associated ASVs to highly predict iron concentration in maize shoots was cross-validated through their performance in random forest (RF) regression. Only the ten families with the highest numbers of ASVs that positively correlated with iron concentration are shown. Taxonomy is color-coded at the phylum level. Following regression, the relative abundances (RA) were summarized (**C** and **D**), and differential relative abundance was performed via pairwise Wilcoxon rank-sum tests. In addition, the abundance of bacterial (**E**) and fungal (**F**) iron-associated ASVs was examined from the families with the highest numbers of iron-associated ASVs (**A** and **B**), i.e., *Comamonadaceae* (bacteria) and *Hypocreaceae* (fungi). Differential abundance was tested via pairwise t-tests. The bars show the RA of differential ASVs, summarized at the genus level. The red dots with error bars depict the mean cumulative RA of these ASVs, and the error bars indicate the standard deviation. Ctrl: Control, BMc: Beneficial microorganism consortium, No.: Number. Different letters indicate significant statistical differences between the groups (Wilcoxon rank-sum or Student’s t-test, *p* < 0.05, Benjamini-Hochberg correction, *n* = 16)

To identify ASVs that correlate with iron uptake, we performed LMs with iron shoot concentrations and ASV log_10_ transformed relative abundances. In total, 239 bacterial and 110 fungal ASVs significantly and positively correlated with shoot iron concentrations (LM, β-coefficient > 0.005, *p* < 0.05, Benjamini-Hochberg correction, Fig. 7A and B, Table S15). Most of the iron-associated bacterial ASVs belonged to *Comamonadaceae* (30 ASVs) followed by *Oxalobacteraceae* (21 ASVs, Fig. 7A). Meanwhile, most of the iron-associated fungal ASVs belonged to *Hypocreaceae* (10 ASVs) followed by *Mortierellaceae* (8 ASVs, Fig. 7B). We further verified the predictability of these ASVs by testing their dependence on farming practice via linear (Fig. S6) and random forest regression (Fig. 7). Based on the random forest algorithm, these fungal and bacterial iron-associated ASVs could explain ~ 50% of the variance of shoot iron concentrations (Fig. 7A and B). Moreover, their cumulative relative abundance significantly increased due to BMc inoculation in 2020 (Wilcoxon rank-sum test, *p* < 0.05) but was generally higher in 2021 (Fig. 7C and D). Furthermore, analyses at genus level indicated that most of the iron-associated *Comamonadaceae* ASVs belonged to *Acidovorax*,* Rhizobacter*,* Caenimonas*, and unclassified genera (Fig. 7E). All fungal iron-associated ASVs of the *Hypocreaceae* were classified as *Trichoderma*, except one ASV classified as *Kiflimonium* (Fig. 7F). In total, the abundance of *Comamonadaceae* and *Hypocreaceae* significantly increased due to BMc inoculation in 2020 (t-test, *p* < 0.05, *n* = 16, Benjamini-Hochberg correction). However, both families showed significantly higher relative abundances in 2021 than in 2020 (t-test, *p* < 0.05, *n* = 16, Benjamini-Hochberg correction), but their relative abundance did not increase due to BMc inoculation.

### Genomes closely related to *Comamonadacea*e ASVs possessed potential drought mitigating traits

**Fig. 8:**
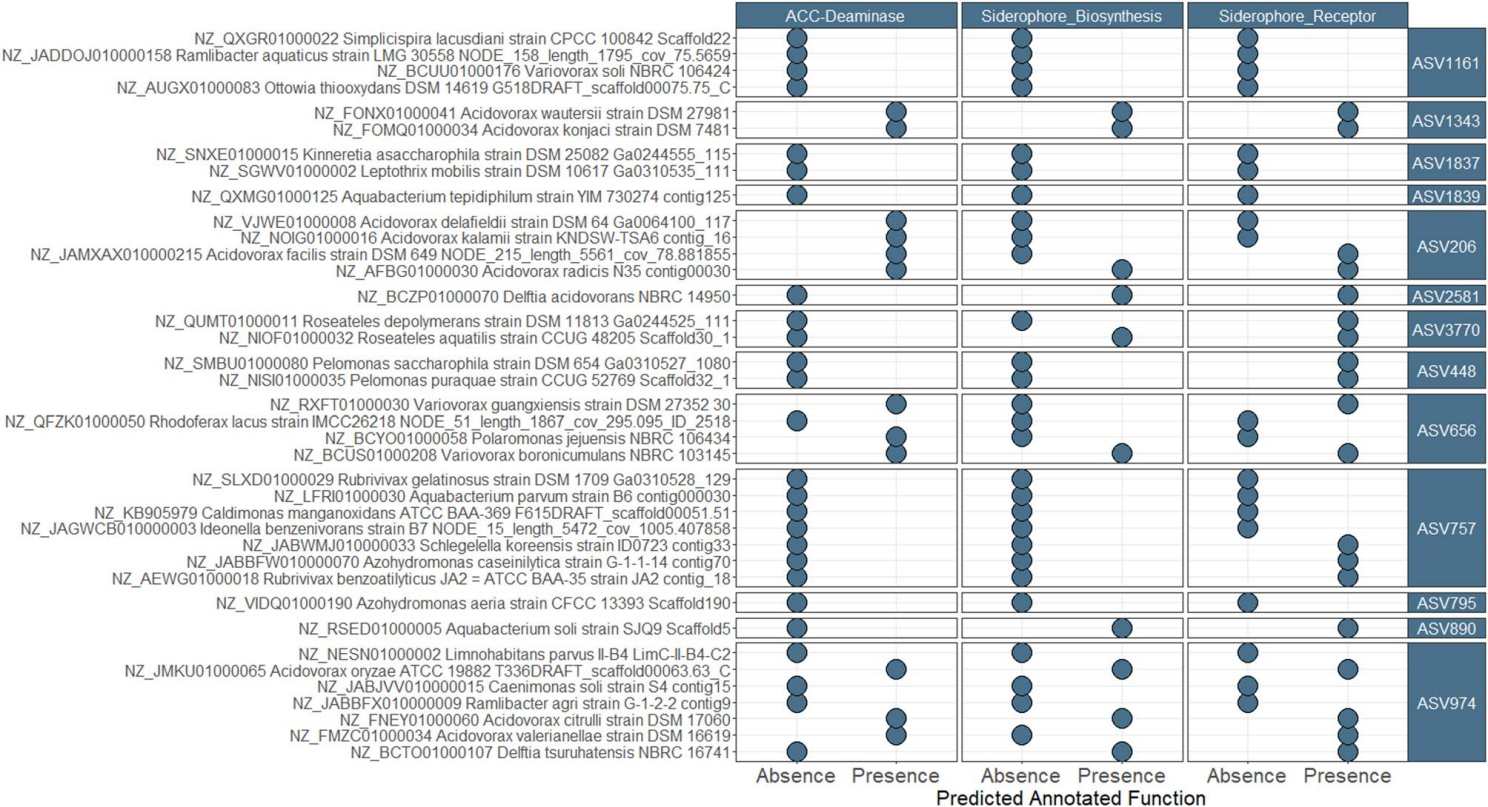
Absence and presence of ACC (1-aminocyclopropane-1-carboxylate) deaminase and predicted siderophore gene or receptor functions in publicly available genomes from the genome taxonomy database (GTDB), which phylogenetically aligned to iron associated *Comamonadaceae* ASVs (min. 97% alignment similarity, Fig. 7A). The genomes were aligned with the ASVs by using usearch. Genomes were annotated with PROKKA to identify ACC-deaminase genes and antiSMASH to find putative siderophore-associated genes

Since metagenome sequencing did not produce fully assembled bacterial genomes, we further aimed to confirm whether the predicted iron-associated bacterial ASVs possessed siderophore genes or drought-responsive traits associated with plant growth due to BMc inoculation. Thus, we analyzed publicly available sequence data and identified 38 genomes that were phylogenetically related to 13 *Comamonadaceae* ASVs (≥ 97% similarity). Of the 38 genomes, ten harbored putative siderophore biosynthesis genes, and 19 contained siderophore receptor genes. Furthermore, twelve genomes displayed putative ACC deaminase functions. Several genomes classified as *Variovorax* and *Acidovorax* showed ACC deaminase and siderophore-associated genes (Fig. 8), indicating their prominent role as resident BMs. These results indicate that the predicted iron-associated ASVs probably belonged to microbial taxa with several crucial plant beneficial traits that could mitigate drought effects.

## Discussion

Inoculation with plant beneficial microorganisms usually shows promising results under controlled lab and greenhouse conditions, but their field performance varies, limiting widespread agricultural use [68, 69]. The reasons for this, particularly the effects of farming practices and environmental factors on rhizosphere competence and functional activity, remain unclear [70, 71]. By using a multi-disciplinary approach and field experiments, we demonstrated that the growing season influenced BMc inoculation effects on maize plants by shaping the rhizosphere microbiome. Our results indicate that microbial inoculants exhibited a complex interplay with maize plants and their resident rhizosphere microbiome under drought. In the following sections, we discuss how our findings enhance the understanding of how different environmental conditions and farming practices can influence the observed effects of microbial inoculants on crop performance at the field scale.

### Establishment of microbial inoculants in the maize rhizosphere

The ability of microbial inoculants to colonize and survive in the rhizosphere is crucial for successfully promoting crop performance [15, 25, 63]. Factors like farming practice, environmental conditions, and competition with native microbes may affect inoculant establishment and thus counteract positive effects on plant performance [64–66]. Environmental factors that can cause disturbances, such as heat or drought, can increase the establishment rates of non-native microbes [65], which agrees with our herein findings. Moreover, in this study, we analyzed whether farming practices (intensity of tillage, pesticide use, and N-fertilization) and variations in weather conditions during two growing seasons affect the colonization ability of a plant beneficial microorganism consortium (BMc: *Bacillus atrophaeus* ABi03, *Pseudomonas* sp. RU47, and *Trichoderma harzianum* OMG16) in the rhizosphere of maize, and thus plant microbial interactions. The single strains of the consortium are known for their beneficial traits, and their rhizosphere establishment under both greenhouse and field conditions has been previously shown [26].

Notably, in 2020, maize plants experienced a severe spring and early summer drought with a much lower precipitation average compared to 2021 (Fig. S4). Interestingly, cultivation-based and molecular methods confirmed the ability (particularly of the bacterial strains) to efficiently colonize the maize rhizosphere, independently of farming practice and weather conditions in the two years, indicating their strong competitiveness in interaction with the native microbiota, which is critical for agricultural use. Drought is supposed to greatly impact soil/rhizosphere microbial communities [65] and typically reduces microbial activity [66]. However, our cultivation-based approach showed that bacterial inoculant density was higher, especially for ABi03, but also for RU47 in 2020 than in 2021, possibly because *Bacillus* spp. are spore-forming bacteria and, therefore, more tolerant to drought. A previous study observed similar microbial taxa that form beneficial interactions with plants under drought through enrichment in the rhizosphere [67]. Moreover, the fungal strain *T. harzianum* OMG16 was less abundant in 2020, likely due to drought, which tends to reduce fungal abundance, particularly mutualistic fungi [68]. While negative interactions between *T. harzianum* OMG16 and the bacterial inoculants cannot be excluded under well-watered conditions, our metagenomics results did not indicate any negative interactions in the rhizosphere for all inoculants in both years.

### The effect of microbial inoculation on crop performance is connected to iron-nutritional status under drought

Although in our study, all inoculated strains colonized the maize rhizosphere independently of farming practice in both years (Fig. 3A), positive effects on maize growth at the flowering stage induced by BMc inoculation were observed only under drought conditions in 2020 (Fig. 1A). This indicated that differences in plant growth between the two years were not due to different abundances of the BMc strains in the rhizosphere. Albeit no nutrient deficiencies [55] were detected in any treatment (Tables S4, S5). BMc inoculation exclusively increased the iron-nutritional status in 2020 (Fig. 1A). Drought is known to limit iron availability in soil [17] and additionally iron acquisition in graminaceous plant species by the inhibition of phytosiderophore-mediated iron mobilization in the rhizosphere [69]. These results suggest that our inoculants may specifically enhance iron uptake under drought conditions. Iron is critical for plant growth, playing a pivotal role in physiological processes like photosynthesis [70], and contributes to cellular mechanisms that mitigate the impact of abiotic stressors, such as activating antioxidative defense systems [71]. Accordingly, a previous study conducted at the same field site revealed increased activities of iron-dependent enzymes involved in ROS (H_2_O_2_) detoxification under drought stress conditions (ascorbate peroxidase, superoxide dismutase) in the leaf tissue of drought-affected BMc-inoculated plants [36]. This was associated with reduced H_2_O_2_ accumulation in leaves, indicating improved ROS detoxification. Moreover, BMc inoculation increased the rhizosphere accumulation of microbial metabolites with the ability to induce drought adaptations in plants (trehalose) and increased various low molecular weight compounds such as phenolic acids, flavonoids, and benzoxazinoids. These compounds are known as components of root exudates with functions in iron mobilization, pathogen and pest defense, and the ability to modify the composition of rhizosphere microbial communities [36]. The increased iron uptake in 2020 in BMc-treated plants coincided with the downregulation of stress-related genes and upregulation of the gene *ZmNAS3* (Fig. 2), which is directly connected with nicotianamine synthesis, a metal-chelating molecule involved in iron metabolism. Consequently, the improved iron status may be linked to enhanced plant stress tolerance. In conclusion, our results revealed that environmental factors can heavily shape the observed effect of BMc inoculation on crop performance and that these observed effects were directly associated with iron-nutritional status.

### Complex interplay between the inoculated microbial strains and the resident rhizosphere microbiota

To explore the connection of microbiota composition with drought effects, we directly correlated bacterial and fungal ASVs with nutrient concentrations in maize shoots (Fig. 7, Table S15). ASVs positively correlated with iron concentration explained up to 50% of the variance in shoot iron concentrations (Fig. 7). These key iron-associated ASVs were specifically enriched in the rhizosphere of BMc inoculated plants in 2020, but exhibited higher overall abundances in 2021 when plants grew under less stressful conditions. Previous studies have shown rhizosphere microbiome composition plays a critical role in iron uptake under drought conditions [17, 66], supporting our herein observed interaction effect between drought and BMc inoculation on maize growth. Due to crop rotation in the experimental design, BMc inoculation in each year was applied to different plots, meaning the presence of these ASVs in 2021 (even in control plots) cannot be attributed to BMc inoculation in 2020. Thus, our results confirm and extend previous findings [17, 66], suggesting that seasonal changes altered plant-microbe interactions in the rhizosphere. Our results indicated that microbial inoculation probably assisted in recruiting resident beneficial microorganisms, especially in the dry year. This aligns with previous findings, which indicated that microbial inoculation could promote the recruitment of beneficial microorganisms from resident soil microbiomes, especially when plants suffer from abiotic stress factors like nutrient limitation [26, 72].

Shotgun metagenomics of the rhizosphere revealed the presence of more plant beneficial gene functions in 2020 compared to 2021 (Fig. 6B). The functional potential of the rhizosphere microbiome to enhance plant beneficial traits is a crucial indicator for sustainable practices that improve plant fitness [35]. In both years, many of the detected genes were also present in the *Bacillus atrophaeus* ABi03 genome. Secondary metabolites, such as surfactin, iturin, and fengycin, primarily involved in biotic interactions [73], likely contributed to the shifts in β-diversity following BMc inoculation in both years. Many of these secondary metabolites can also act as siderophores, chelating iron, and can play a vital role in iron uptake by plants [74], pathogen suppression through iron competition [75], and the induction of systemic plant resistance [74, 76]. Siderophore production is widely recognized as a beneficial microbial function [17], potentially improving plant iron acquisition [74], and may have contributed to the enhanced iron concentration in plant tissue and overall plant performance observed in the present study.

Moreover, we were able to confirm the presence of siderophores in publicly available genomes from the *Comamonadaceae* family, which were phylogenetically aligned to the herein iron-associated ASVs, using antiSMASH, which utilizes Hidden Markov Models [62] to predict biosynthetic clusters, including siderophore clusters. This family, commonly found in soil and rhizosphere communities, includes critical genera such as *Acidovorax* and *Variovorax*, both known for their plant beneficial traits [21]. These findings support previous studies showing that *Comamonadaceae*, particularly *Variovorax*, can promote root growth and alleviate the impact of drought on plants [21, 77]. Members of *Variovorax* frequently possess genes for ACC deaminase and siderophore biosynthesis [21, 78]. In addition, our study revealed insights into iron-associated ASVs within the fungal family *Hypocreaceae*. Despite the absence of fungal genomes for analysis in GTDB [59], *Hypocreaceae* members, such as *Trichoderma* spp., have a wide range of plant beneficial characteristics and are used as bio-stimulants [38, 79]. Since BMc inoculation significantly increased resident *Trichoderma* ASVs, this indicates the enrichment of beneficial fungi in the rhizosphere microbiome of maize. These results further suggest that the iron-associated taxa predicted by our modeling approach possess plant beneficial characteristics.

These results indicate a complex interplay between resident rhizosphere microorganisms and the inoculated BMc strains. However, we could not disentangle whether this complex interplay was mediated by direct (microbe-microbe) or indirect interactions. For example, we cannot exclude that the BMc strains affected rhizosphere microorganisms through interfering with the plant exudation patterns by increasing the production of phytosiderophores, which could impact the availability of iron for microorganisms and hereby caused the observed shifts in β-diversity. To mechanistically disentangle whether rather a direct or indirect modulation of the microbiome by BMc strains took place, it requires experiments under controlled laboratory conditions coupled with stable isotope probing and meta-transcriptomics to capture nutrient flows, along with plant-metabolomics of gnotobiotic experiments for gaining insights into the impact of microbial inoculants on root exudation profiles. Future research should focus on how various levels of drought affect these hypothesized direct and indirect interactions between inoculated and resident microorganisms and by which mechanisms they are mediated (e.g., exudation stimulation or microbial cross-feeding).

### Farming practice effects on the rhizosphere microbiome and crop performance

Farming practices and environmental conditions can play a significant role in shaping microbiome composition as ecological processes through selective pressures [80, 81] and neutral stochastic processes (e.g., dispersion) [82]. Tillage, in particular, had a pronounced impact on bacterial and fungal community assembly, confirming previous studies [34, 35, 50, 83, 84]. Intensive tillage practices change soil structure and create new conditions and niches for microorganisms and increase the dispersion of microbial taxa [80, 81]. These effects probably explain the strong influence of tillage on rhizosphere microbiome composition observed in this study. Surprisingly, our study revealed that the impact of different farming practices, such as tillage and N-fertilization intensity, on the observed effects of BMc inoculation on maize performance was minor. In contrast, we found that environmental conditions, such as drought, had a much stronger influence than farming practices on the effect of microbial inoculants on improving maize growth, and the bacterial inoculants completely overcame the drought stress. These findings agree with previous studies, which indicated the weaker influence of fertilization on inoculation effects on plants compared to environmental factors [26, 85, 86, 87].

## Conclusion

In summary, our study revealed that mainly precipitation amounts determined the effects of microbial inoculants on maize performance, and these effects were connected to iron uptake under drought. Overall, drought did not reduce the persistence of the individual bacterial BMc strains but affected the persistence of the fungal inoculant strain, strongly reshaping the rhizosphere microbiome and taxa responding to BMc inoculation. Consequently, microbial inoculation outcomes varied depending on drought, and this effect was connected with shifts of specific microbial taxa due to BMc inoculation, which were also associated with iron uptake. Our results indicated a complex interplay between inoculated plant beneficial and resident rhizosphere microorganisms, since the microbial inoculants enriched the abundance of specific microbial taxa under drought. Therefore, our findings emphasize the importance of the complex interactions among microbial inoculants, the resident rhizosphere microbiome, and plants (particularly under abiotic stress factors) for the inoculation outcome on crop performance. A holistic approach is crucial for understanding how microbial inoculants can positively affect microorganisms with plant beneficial functions in drought-impacted ecosystems and whether these effects are mediated through direct microbe-microbe interactions and/or indirectly via plant-microbe interactions. In conclusion, selecting beneficial microbial inoculants should account for their performance under varying environmental conditions.

## Electronic supplementary material

Below is the link to the electronic supplementary material.

Supplementary Material 1

Supplementary Material 2

## Data Availability

Sequencing data (16S rRNA gene amplicon and shotgun) were deposited at the Sequence Read Archive (https://www.ncbi.nlm.nih.gov/sra) under the BioProject accession PRJNA1045550. Fungal ITS2 sequences can be found at the European Nucleotide Archive (ENA) under PRJEB74508. Raw plant data can be found in the BONARES repository (https://doi.org/10.20387/bonares-w669-gdsd). Additional datasets and the scripts used for this study can be found in the https://github.com/JonKampouris/Bernburg_BMc_LTE2Y.
