# Supplementary material for: Selective recruitment of beneficial microbes in the rhizosphere of maize affected by microbial inoculants, farming practice, and seasonal variations
Source: Environ Microbiome. 2025 Jun 12;20:69. doi: 10.1186/s40793-025-00729-y (PMC12164104; doi:10.1186/s40793-025-00729-y)
Supplement: Supplementary file 1 — Supplementary Material 1 [file 40793_2025_729_MOESM1_ESM.docx]

***Supplementary Material***

**Selective recruitment of beneficial microbes in the rhizosphere of maize affected by microbial inoculants, farming practice, and seasonal variations**

**Ioannis D. Kampouris^1*#^, Theresa Kuhl-Nagel^2#^, Jan Helge Behr^2^, Loreen Sommermann^3^, Doreen Babin^1^, Davide Francioli^4,5^, Rita Zrenner^2^, Susanne Kublik^6^, Michael Schloter^6,7^, Uwe Ludewig^4^, Kornelia Smalla^1^, Günter Neumann^4^, Rita Grosch^2#^, Joerg Geistlinger^3#^**

^1^Julius Kühn Institute (JKI) - Federal Research Centre for Cultivated Plants, Institute for Epidemiology and Pathogen Diagnostics, Braunschweig, Germany

^2^Leibniz Institute of Vegetable and Ornamental Crops (IGZ), Plant-Microbe Systems, Großbeeren, Germany

^3^Department of Agriculture, Ecotrophology and Landscape Development, Anhalt University of Applied Sciences, Bernburg, Germany

^4^Department of Nutritional Crop Physiology, Institute of Crop Science, University of Hohenheim, Stuttgart, Germany

^5^Department of Soil Science and Plant Nutrition, Hochschule Geisenheim University, Geisenheim, Germany

^6^Institute for Comparative Microbiome Analysis, Helmholtz Zentrum München – German Research Center for Environmental Health, Neuherberg, Germany

^7^Technical University of Munich, Chair for Environmental Microbiology, München, Germany

^#^ I. D. Kampouris, T. Kuhl-Nagel, R. Grosch, J. Geistlinger contributed equally to this work

*** Correspondence:**Ioannis D. Kampouris; ioannis.kampouris@julius-kuehn.de

**Table S1** Genes and primer pairs for gene expression analysis in maize leaves (cv. Benedictio). Housekeeping genes for normalization are highlighted in bold.

| **Gene** | **Code** | **Accession** | **Forward and**  **Reverse primers (5’-3’)** | **Ref** |
| --- | --- | --- | --- | --- |
| **Ubiquitin-conjugating enzyme** | *ZmUbi* | NM_001154750.1 | CAGGTGGGGTATTCTTGGTG  ATGTTCGGGTGGAAAACCTT | 1 |
| **Actin** | *ZmACT* | NM_001165684.1 | GGAGCTCGAGAATGCCAAGAGCAG  GACCTCAGGGCATCTGAACCTCTC | 2 |
| Cytosolic ascorbate peroxidase | *ZmAPX1* | GRMZM2G316256 | GCTCTGTCTTGCATGGCACTCC  GATGGGCTCTAGCAACCTGACG | 3 |
| Mitogen-activated protein kinase | *ZmMPK3* | GRMZM2G017792 | ACAGCGACATGATGACGGAGTA  CCAATCACCTCGGTTATGAG | 3 |
| Nicotianamine synthase | *ZmNAS3* | XM_008666956.4 | GGCTCACCAGAAGATGGAGGAG  TCACGCATGTGGTGTAGACACG | 4 |
| Zinc transporter | *ZmIRTa* | NM_001158638.2 | CTGCAGAGCAGCGTCAGG  AGTACTGTGTCATGTCGTCTC | 5 |
| Oligopeptide transporter | *ZmOPT8a* | GRMZM2G086258 | GCTACATGAGCATGTCGCAGGCT  TGCCAGCCACAATGGTACCAACAAACTGA | 5 |
| Nitrite reductase | *ZmNIR* | GRMZM2G079381 | AGGTGGCGGACATCGGCTTC  ACGGCACGGACTTCCTGTAGAC | 6 |
| Nitrate reductase 1 | *ZmNR1* | GRMZM2G076723 | TGCTTCTGGTCCGTCGAGGTGG  ACACGTTCACCTTCACCTTGAA | 7 |
| High affinity nitrate transporter | *ZmNAR2.2* | GRMZM2G163494 | GCTGGAGGTGACCCTCTGCTACG  TGCCGGGCGATCCTGAACTGG | 7 |
| Phosphate transporter 1;1 | *ZmPht1* | GRMZM2G326707 | CGTAGTACGTGTGTGATAGTCTGG  TATTATCACACGTGGACCTCTACC | 8 |
| Phosphate transporter 1;3 | *ZmPht3* | GRMZM2G112377 | GCCTTCCGTTACGTCATTGT  AGCACGTCTCTGATCCCATC | 8 |
| Phosphate transporter 1;4 | *ZmPht4* | GRMZM2G170208 | ACCGGCTACCCTCACCTACT  CTACCTTCTTGGCGTCCTTG | 8 |
| Phosphate transporter 1;8 | *ZmPht8* | GRMZM2G045473 | CCTGGAGGAGATGTTCAGGA  AAGACGGTGAACCAGTAGCC | 8 |
| Endochitinase PR4 | *ZmPR4* | NM_001157282.1 | TGATGGATAGATGGCGATTGC  AGAATTGACACCGCCAAACC | 9 |
| Superoxide dismutase | *ZmSOD4* | XM_008653632.4 | CACCAACGGCTGCATGTC  ATGCTCCTTGCCAACAGGAT | 9 |
| 1-Aminocyclopropane-1-carboxylate synthase | *ZmACS6* | NM_001143622.2 | GTGCTCATCACCAACCCTTC  ACGAAGTCCACCAGCATCTC | 2 |
| Defensin-like protein 1 | *ZmDef1* | NM_001329491.1 | TGCTGCTCCTCATCGTCGTTGC  TTGCCGCCGCCGTAGCCTTC | 10 |
| Defensin-like protein 2 | *ZmDef2* | NM_001153491.2 | AGTCCAGGGCGACCGTGTG  CGAGTGGTGCTGGCTCTTGC | 10 |
| WRKY transcription factor | *ZmWRKY106* | GRMZM2G013391 | GCTCGTCACCTACACCTTCG  AGCTTTCGTCCTCCTCTGC | 11 |
| WRKY transcription factor | *ZmWRKY17* | GRMZM2G102583 | TTTTTCTTCTTCCGCTGTTCTACTC  TCAGATCGAGGGTCGTCATCT | 11 |
| WRKY transcription factor | *ZmWRKY33* | GRMZM2G148087 | GTGGTCCAGACGATGAGCGACAT  GCTGCTCAGCATCTCCAGGGTGT | 11 |
| WRKY transcription factor | *ZmWRKY58* | GRMZM2G147880 | AGGAAGTGGAGGAGGCGAACA  GGATGGCTTGCGCTTGC | 11 |
| Ethylene-responsive factor-like protein | *ZmERF1* | NM_001111800.2 | ACTTCCCCAGCGACACCTC  TGACCTCGTCGGACACCTGA | 12 |
| Ethylene-responsive transcription factor | *ZmEREB58* | NM_001176924.1 | GACGGCGACAAGAAGCGA  CGGTGCCAGGACGACG | 13 |
| MYB transcription factor | *ZmMYB30* | GRMZM2G087955 | CTCCTTGTCGTTGTCCCTCT  CTTGCTTGTGCTTGAGGTGT | 14 |
| MYB transcription factor | *ZmMYB36* | GRMZM2G139284 | GGTGTTCGAGTACGAGACGA  ACAGGACGGTGGAAGTGG | 14 |
| MYB transcription factor | *ZmMYB95* | GRMZM2G139284 | CTCGTCTTCTCTCCGCTACC  TAGTCGACGACAACGAGTGG | 14 |

**References**

1 Gorbani et al., 2018; https://doi.org/10.1371/journal.pone.0194592

2 Louis et al., 2015; https://doi.org /10.1104/pp.15.00958

3 Xu et al., 2017; http://dx.doi.org/10.1016/j.plaphy.2017.05.018

4 Zhou et al. 2013; http://www.biomedcentral.com/1471-2164/14/238

5 Kobae et al., 2014; http://dx.doi.org/10.1080/00380768.2014.949854

6 Jiang et al., 2018; https://doi.org/10.1080/23723556.2018.1441633

7 Liseron-Monfils et al., 2013; https://doi.org/10.4161/psb.26056

8 Sawers et al., 2017; https://doi.org/10.1111/nph.14403

9 Gond et al., 2015; http://dx.doi.org/10.1016/j.micres.2014.11.004

10 Liu et a., 2016; https://doi.org/10.1093/jxb/erw226

11 Wei et al., 2012; https://doi.org/10.1093/dnares/dsr048

12 Shi et al., 2016; https://doi.org/10.1007/s11738-016-2146-2

13 Li et al., 2015; https://doi.org/10.1111/tpj.12994

14 Chen et al., 2018; https://doi.org 10.1007/s10535-017-0756-1

**Table S2** Information on metagenomic sequencing runs (AR: annotated reads)

|  | **2020** | | | | **2021** | | | |
| --- | --- | --- | --- | --- | --- | --- | --- | --- |
|  | **No. of reads** | **Range** | | **[%]** | **No. of reads** | **Range** | | **[%]** |
| **Raw reads (Forward & Reverse)** | 755,064,346 | 7,694,159 – 19,737,260 | | 100 | 720,503,706 | 9,266,032 – 14,941,948 | | 100 |
| **Trimmed reads (Forward & Reverse) using FASTX toolkit5 (50bp, Phredscore 20, No PhiX contamination, paired)** | 720,371,138 | 7,352,763 – 18,813,545 | | 95.4 | 693,723,530 | 8,993,359 – 14,472,065 | | 96.3 |
| **Merged reads by PEAR pipeline** | 344,628,088 | 7,168,494 – 18,104,610 | | 91.3 | 321,676,174 | 7,885,933 – 13,151,655 | | 89.3 |
| **Merged read length ranges** |  | 120-165 bp | |  |  | 119-134 bp | |  |
| **Taxonomy merged reads by MGX pipeline** | **2020** | | | | **2021** | | | |
|  | **AR** | | **AR [%]** | | **AR** | | **AR [%]** | |
| Archaea | 2,762,345 | | 0.8 | | 2,556,190 | | 0.8 | |
| Bacteria | 152,802,549 | | 44 | | 156,554,970 | | 49 | |
| Fungi | 124,178 | | 0.04 | | 202,646 | | 0.06 | |
| Total (also includes other eukaryotes) | 156,817,662 | | 45.5 | | 160,603,667 | | 50 | |
| **Functions merged reads (COG database)** | **AR** | | **AR [%]** | | **AR** | | **AR [%]** | |
| Archaea | 1,151,439 | | 0.3 | | 1,129,602 | | 0.35 | |
| Bacteria | 92,086,588 | | 25.5 | | 95,164,223 | | 30 | |
| Fungi | 46,384 | | 0.012 | | 76,561 | | 0.02 | |
| Total (also includes other eukaryotes) | 100,997,321 | | 28 | | 101,883,650 | | 31.7 | |

**Table S3** Linear model with the factors affecting shoot biomass (SDM) of maize (cv. Benedictio). Log_10_ transformation was applied to improve fitting under normal distribution. Significant factors are highlighted in bold, n = 64. Shapiro test on residuals using log_10_-transformed data *p* = 0.01, n = 64.

| **Parameter** | **R^2^** | **F** | ***p*-value** |
| --- | --- | --- | --- |
| **BMc Inoculation** | 7.20 | 7.461929 | 0.00879 |
| Year | 0.07 | 0.067868 | 0.79558 |
| Tillage | 0.31 | 0.319058 | 0.57480 |
| **N-Fertilization intensity** | 4.68 | 4.851645 | 0.03245 |
| **BMc Inoculation × Year** | 21.93 | 22.73355 | 0.00002 |
| **BMc Inoculation × Tillage** | 11.45 | 11.86324 | 0.00120 |
| Year:Tillage | 1.90 | 1.96771 | 0.16713 |
| BMc Inoculation × N-Fertilization intensity | 0.01 | 0.01319 | 0.90905 |
| Year × N-Fertilization intensity | 0.42 | 0.434849 | 0.51277 |
| Tillage × N-Fertilization intensity | 1.06 | 1.098288 | 0.29989 |
| BMc Inoculation × Year:Tillage | 0.15 | 0.151103 | 0.69920 |
| **BMc Inoculation × Year × N-Fertilization intensity** | 4.06 | 4.211864 | 0.04562 |
| BMc Inoculation × Tillage × N-Fertilization intensity | 0.11 | 0.111118 | 0.74033 |
| Year × Tillage × N-Fertilization intensity | 0.24 | 0.252669 | 0.61750 |
| BMc Inoculation × Year × Tillage × N-Fertilization intensity | 0.10 | 0.108665 | 0.74310 |

**Table S4**: The table depicts main and interactions effects of ANOVA tests on the macro- and micro-nutrient concentrations in maize shoots (cv. Benedictio) grown under different farming practices: Mouldboard plough tillage or Cultivator tillage (TILL), Intensive or Extensive N-fertilization intensities (FERT), and with or without BMc (beneficial microorganism consortium) inoculation. The table depicts data from maize plants in the growing season 2020 (**p* < 0.05, ***p* < 0.01, ****p* < 0.001, n=64).

|  | **C** | **N** | **P** | **K** | **Mg** | **Ca** | **Fe^#^** | **Cu** | **Zn** | **Mn^#^** |
| --- | --- | --- | --- | --- | --- | --- | --- | --- | --- | --- |
| TILL | 0.058 | 0.017* | < 0.001*** | 0.023* | 0.009** | 0.947 | 0.003** | 0.021* | 0.016* | 0.012* |
| FERT | 0.853 | 0.005** | < 0.001*** | 0.503 | < 0.001*** | 0.055 | 0.929 | 0.003** | 0.551 | 0.164 |
| BMc Inoculation | 0.034* | 0.157 | 0.121 | 0.562 | 0.151 | 0.938 | 0.008** | 0.060 | 0.848 | 0.815 |
| TILL x FERT | 0.802 | 0.575 | 0.036* | 0.371 | 0.562 | 0.131 | 0.038* | 0.742 | 0.193 | 0.815 |
| TILL x BMc Inoculation | 0.386 | 0.376 | 0.654 | 0.910 | 0.909 | 0.387 | 0.045* | 0.976 | 0.617 | 0.519 |
| FERT x BMc Inoculation | 0.295 | 0.622 | 0.400 | 0.427 | 0.004** | 0.468 | 0.572 | 0.142 | 0.834 | 0.664 |
| TILL x FERT x BMc Inoculation | 0.223 | 0.838 | 0.985 | 0.505 | 0.198 | 0.188 | 0.428 | 0.415 | 0.544 | 0.596 |

**Table S5**: The table depicts main and interactions effects of ANOVA tests on the macro- and micro-nutrient concentrations in maize shoots (cv. Benedictio) grown under different farming practices: Mouldboard plough tillage or Cultivator tillage (TILL), Intensive or Extensive N-fertilization intensities (FERT), and with or without BMc (beneficial microorganism consortium) inoculation. The table depicts data from maize plants in the growing season 2021 (**p* < 0.05, ***p* < 0.01, ****p* < 0.001, n=64).

|  | **C** | **N** | **P** | **K** | **Mg** | **Ca** | **S^#^** | **Fe** | **Cu** | **Zn** | **Mn** |
| --- | --- | --- | --- | --- | --- | --- | --- | --- | --- | --- | --- |
| TILL | 0.001** | < 0.001*** | 0.118 | 0.018* | 0.002** | < 0.001*** | < 0.001*** | 0.006** | < 0.001*** | < 0.001*** | < 0.001*** |
| FERT | 0.055 | < 0.001*** | 0.010* | 0.063 | < 0.001*** | 0.524 | < 0.001*** | 0.118 | 0.367 | 0.207 | < 0.001*** |
| BMc Inoculation | 0.985 | 0.772 | 0.198 | 0.700 | 0.802 | 0.645 | 0.042* | 0.298 | 0.073 | 0.133 | 0.666 |
| TILL x FERT | 0.637 | 0.024* | 0.006** | 0.009** | 0.455 | 0.111 | 0.433 | 0.956 | 0.006** | 0.523 | 0.245 |
| TILL x BMc Inoculation | 0.304 | 0.935 | 0.695 | 0.009** | 0.130 | 0.126 | 0.093 | 0.699 | 0.264 | 0.701 | 0.557 |
| FERT x BMc Inoculation | 0.408 | 0.018* | 0.454 | 0.803 | 0.346 | 0.335 | 0.470 | 0.230 | 0.037* | 0.798 | 0.531 |
| TILL x FERT x BMc Inoculation | 0.307 | 0.002** | 0.535 | 0.946 | 0.256 | 0.215 | 0.225 | 0.159 | 0.145 | 0.701 | 0.638 |

**Table S6** Statistics of structural equation model (SEM) with SDM (shoot dry mass) and nutrient concentrations (mg or g kg^-1^ SDM) based on partial least squares.

| **Variable1** | **Variable2** | **Estimate** | **p-value** |
| --- | --- | --- | --- |
| SDM | Fe | 2.377422 | 0.017434 |
| SDM | Ca | -1.96555 | 0.049351 |
| SDM | Zn | 2.603525 | 0.009227 |
| SDM | K | 2.531854 | 0.011346 |
| SDM | Cu | -4.17656 | 2.96E-05 |
| Fe | N | 2.803724 | 0.005052 |
| Fe | Ca | 4.851634 | 1.22E-06 |
| Fe | S | 3.302182 | 0.000959 |
| Fe | P | -2.20836 | 0.027219 |
| N | K | 2.206329 | 0.027361 |
| N | Cu | 5.74895 | 8.98E-09 |
| Mg | Ca | 5.248275 | 1.54E-07 |
| Mg | Mn | -10.1431 | 0 |
| Mg | S | 2.307002 | 0.021055 |
| Mg | P | -2.03849 | 0.041501 |
| Mg | Zn | 6.513073 | 7.36E-11 |
| Mg | K | 5.334574 | 9.58E-08 |
| Ca | Mn | 5.36365 | 8.16E-08 |
| Ca | S | -3.5304 | 0.000415 |
| Mn | S | 2.417993 | 0.015606 |
| Mn | P | 4.023389 | 5.74E-05 |
| Mn | K | -9.92839 | 0 |
| Mn | Cu | 3.309991 | 0.000933 |
| P | Zn | 4.234146 | 2.29E-05 |
| P | K | 7.502933 | 6.24E-14 |
| P | Cu | -5.67227 | 1.41E-08 |
| Zn | Cu | 6.491075 | 8.52E-11 |
| K | Cu | 10.42701 | 0 |

**Table S7** Number (No.) of annotated reads (AR, 100%) and sequences identical to the inoculated strains (ABi03, RU47, and OMG16) of the beneficial microorganism consortium in the growing seasons 2020 and 2021.

| **2020** | **AR total** | **No. of AR mapped onto respective BMc genome (identity 100%, alignment 100%)** | **Genome** |
| --- | --- | --- | --- |
| *Bacillus atrophaeus* | 71,345 | 59,164 | ABi03 |
| *Pseudomonas* sp. | 23,221 | 17,462 | RU47 |
| *Trichoderma harzianum* | 1,496 | 474 | OMG16 |
| **2021** |  |  |  |
| *Bacillus atrophaeus* | 125,375 | 106,080 | ABi03 |
| *Pseudomonas* sp. | 24,221 | 19,479 | RU47 |
| *Trichoderma harzianum* | 9,133 | 6,695 | OMG16 |

**Table S8** Global PERMANOVA for bacterial communities in the rhizosphere of control or BMc (beneficial microorganism consortium) inoculated maize plants (cv. Benedictio) under intensive or extensive N-fertilization intensity with mouldboard plough or cultivator tillage, including year as a factor (****p <* 0.001, ***p <* 0.01, **p <* 0.05). Significant factors are highlighted in bold.

|  | Explained Variance [%] | *p*-value |
| --- | --- | --- |
| **Year** | 20.93 | 1.00E-05 |
| **Tillage** | 5.28 | 2.00E-05 |
| N-Fertilization Intensity | 1.65 | 0.07189 |
| **BMc Inoculation** | 2.77 | 0.00433 |
| **Year × Tillage** | 2.17 | 0.0193 |
| Year × N-Fertilization Intensity | 1.26 | 0.21923 |
| Tillage × N-Fertilization Intensity | 1.31 | 0.19293 |
| Year × BMc Inoculation | 1.40 | 0.14837 |
| **Tillage × BMc Inoculation** | 1.99 | 0.03014 |
| N-Fertilization Intensity × BMc Inoculation | 1.40 | 0.14667 |
| Year × Tillage × N-Fertilization Intensity | 1.45 | 0.12775 |
| Year × Tillage × BMc Inoculation | 1.60 | 0.08344 |
| Year × N-Fertilization Intensity × BMc | 1.24 | 0.23698 |
| **Tillage × N-Fertilization Intensity × BMc Inoculation** | 1.81 | 0.04918 |
| Year × Tillage × N-Fertilization Intensity × BMc Inoculation | 1.44 | 0.13044 |

**Table S9** Global PERMANOVA for fungal communities in the rhizosphere of control or BMc (beneficial microorganism consortium) inoculated maize plants (cv. Benedictio) under intensive or extensive N-fertilization intensity with mouldboard plough or cultivator tillage, including year as a factor (****p <* 0.001, ***p <* 0.01, **p <* 0.05). Significant factors are highlighted in bold.

|  | Expl. Variance (%) | *p*-value |
| --- | --- | --- |
| **Year** | 19.42 | 1.00E-05 |
| **Tillage** | 1.90 | 0.00783 |
| **N-Fertilization Intensity** | 4.24 | 1.00E-05 |
| **BMc Inoculation** | 11.49 | 1.00E-05 |
| **Year × Tillage** | 3.53 | 7.00E-05 |
| **Year × N-Fertilization Intensity** | 4.40 | 2.00E-05 |
| **Tillage × N-Fertilization Intensity** | 1.98 | 0.00587 |
| **Year × BMc Inoculation** | 13.59 | 1.00E-05 |
| Tillage × BMc | 0.96 | 0.14752 |
| N-Fertilization Intensity × BMc Inoculation | 0.76 | 0.2851 |
| **Year × Tillage × N-Fertilization Intensity** | 1.80 | 0.01045 |
| Year × Tillage × BMc | 1.04 | 0.11239 |
| Year × N-Fertilization Intensity × BMc | 0.81 | 0.2515 |
| **Tillage × N-Fertilization Intensity × BMc** | 1.31 | 0.04695 |
| Year × Tillage × N-Fertilization Intensity × BMc | 1.17 | 0.07544 |

**Table S10** PERMANOVA for bacterial communities in the rhizosphere of control or BMc (beneficial microorganism consortium) inoculated maize plants (cv. Benedictio) under intensive or extensive N-fertilization intensity with mouldboard plough or cultivator tillage in the sampling years 2020 and 2021 (****p <* 0.001, ***p <* 0.01, **p <* 0.05).

| **Factor** | **Explained Variance [%]** | |
| --- | --- | --- |
|  | **2020** | **2021** |
| **Tillage practice** | 10.0*** | 9.7*** |
| **N-fertilization intensity** | 3.9* | 3.5* |
| **BMc Inoculation** | 5.0*** | 6.8** |
| **Tillage practice ×  N-fertilization intensity** | 3.6 | 3.4 |
| **Tillage practice × BMc Inoculation** | 4.9*** | 4.5** |
| **N-fertilization intensity × BMc Inoculation** | 3.7* | 3.1 |
| **Tillage practice ×  N-fertilization intensity × BMc Inoculation** | 4.6** | 3.7* |
| **Residuals** | 63.2 | 65.2 |

**Table S11** PERMANOVA for fungal communities in the rhizosphere of control or BMc (beneficial microorganism consortium) inoculated maize plants (cv. Benedictio) under intensive or extensive N-fertilization intensity with mouldboard plough or cultivator tillage in the sampling years 2020 and 2021 (****p* < 0.001, ***p <*0.01, **p <* 0.05).

| **Factor** | **Explained Variance [%]** | |
| --- | --- | --- |
|  | **2020** | **2021** |
| **Tillage practice** | 5.9* | 7.1** |
| **N-fertilization intensity** | 3.8* | 14.7*** |
| **BMc Inoculation** | 28.1**** | 32.8**** |
| **Tillage practice ×  N-fertilization intensity** | 7.3** | 3.2** |
| **Tillage practice × BMc Inoculation** | 5.9** | 0.5 |
| **N-fertilization intensity × BMc Inoculation** | 2.5 | 1.7 |
| **Tillage practice ×  N-fertilization intensity × BMc Inoculation** | 5.9** | 1.4 |
| **Residuals** | 40.4 | 38.6 |

**Table S12** Bacterial differentially abundant ASVs to BMc inoculation identified with logistic regression

*See Excel file*

**Table S13** Fungal differentially abundant ASVs to BMc inoculation identified with logistic regression

*See Excel file*

**Table S14** PERMANOVA of bacterial gene abundance from the rhizosphere metagenomes of control or BMc (beneficial microorganism consortium) inoculated maize plants (cv. Benedictio) under intensive or extensive N-fertilization intensity with mouldboard plough or cultivator tillage in the sampling years 2020 and 2021 (****p* < 0.001, ***p <*0.01, **p <*0.05).

| **Factor** | **Explained Variance [%]** | |
| --- | --- | --- |
|  | **2020** | **2021** |
| **Tillage practice** | 7.3** | 6.4*** |
| **N-fertilization intensity** | 4.7* | 3.5 |
| **BMc Inoculation** | 9.0*** | 10.4*** |
| **Tillage practice ×  N-fertilization intensity** | 2.9 | 3.5 |
| **Tillage practice × BMc Inoculation** | 3.4 | 5.7** |
| **N-fertilization intensity × BMc Inoculation** | 3.3 | 4.0* |
| **Tillage practice ×  N-fertilization intensity × BMc Inoculation** | 5.9** | 3.5 |
| **Residuals** | 63.6 | 62.9 |

**Table S15** Bacterial and fungal iron-associated ASVs to BMc inoculation identified with linear regression of iron shoot concentrations and log_10_ transformed relative abundance.

*See Excel file*

**
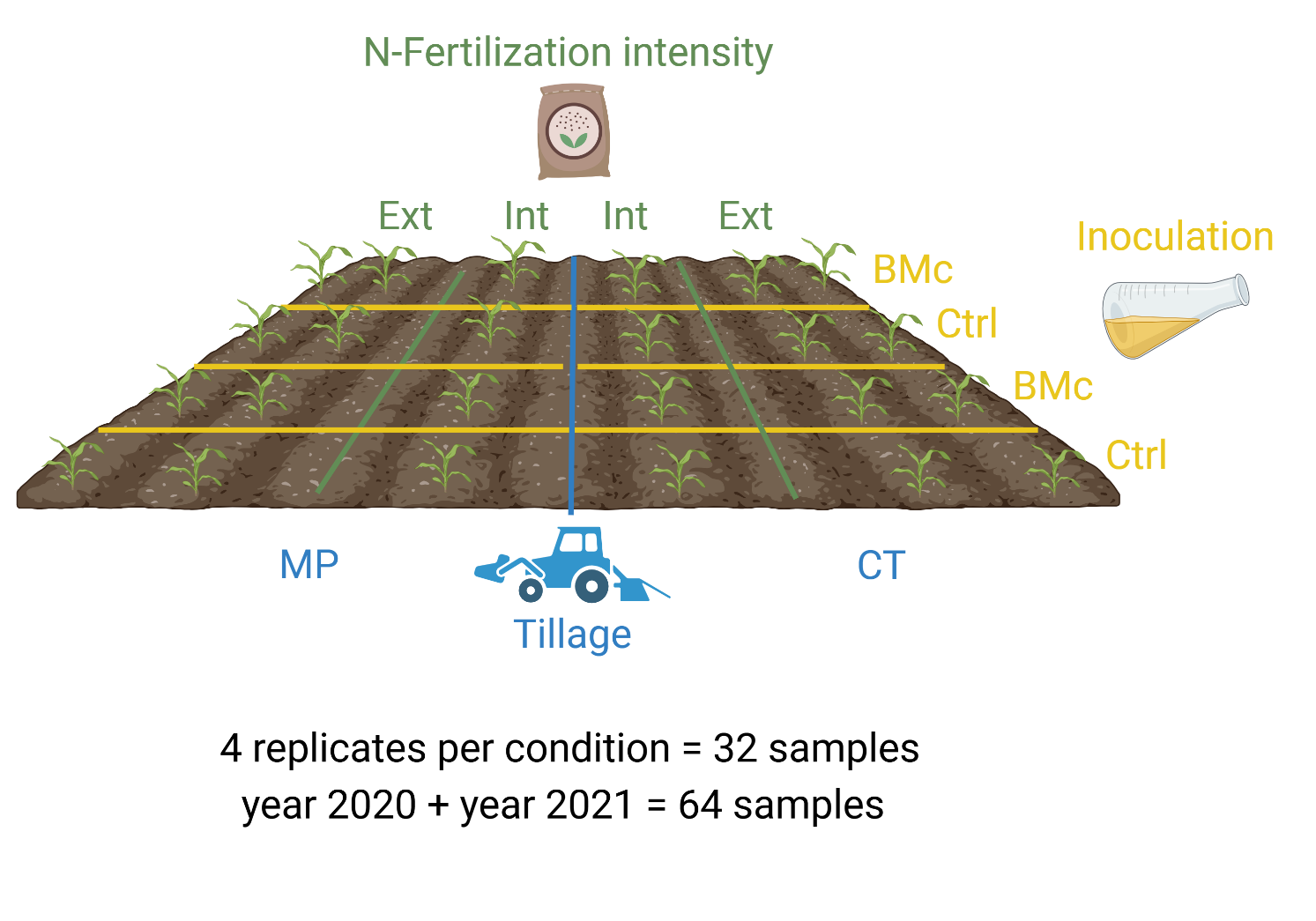
**

**Figure S1.** Description of the experimental design and the number of replicates under different farming practices. BMc: beneficial microorganism consortium, C: Control, Ext: Extensive N-fertilization, Int: Intensive N-fertilization. MP: Mouldboard plough, CT: Cultivator. Created with BioRender.com

**
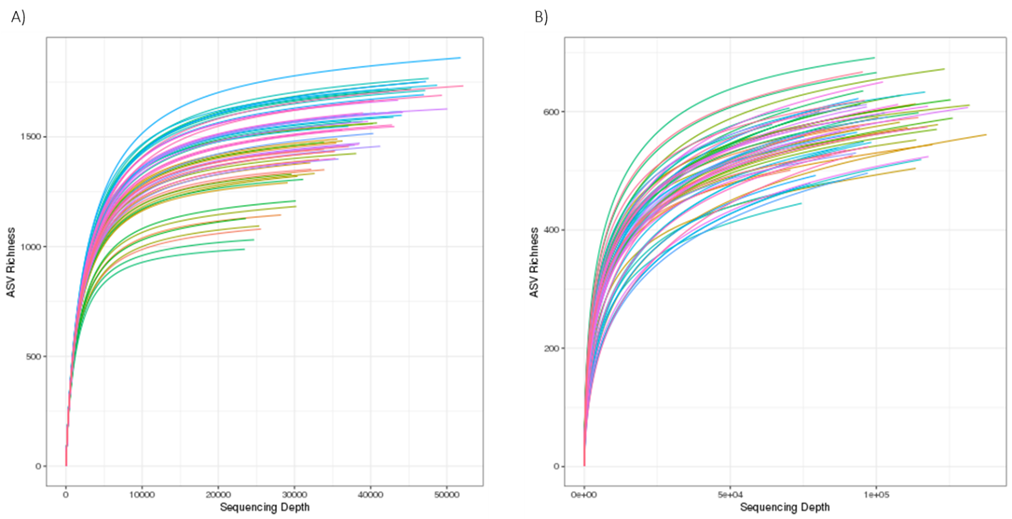
**

**Figure S2.** Rarefaction curves of 16S rRNA gene (A) and ITS2 (B) amplicon sequencing data in the growing seasons 2020 and 2021.

**
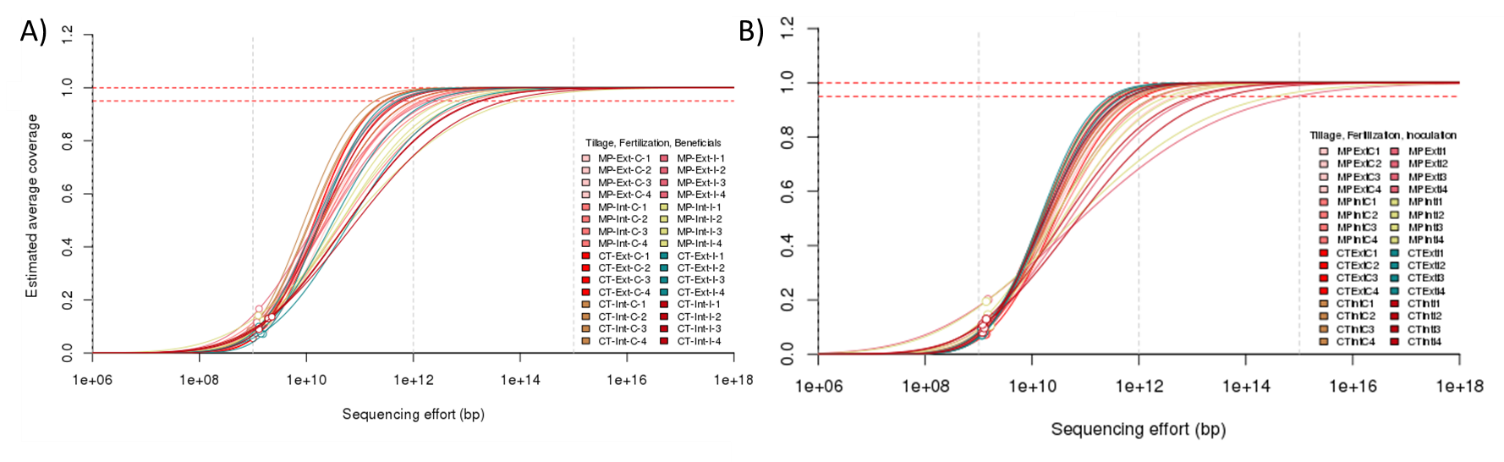
Figure S3.** Nonpareil-curves for diversity coverage of metagenome sequence reads. A) Coverage 2020: 5.4 - 14.5%, B) Coverage 2021: 5.3 - 16.9%.


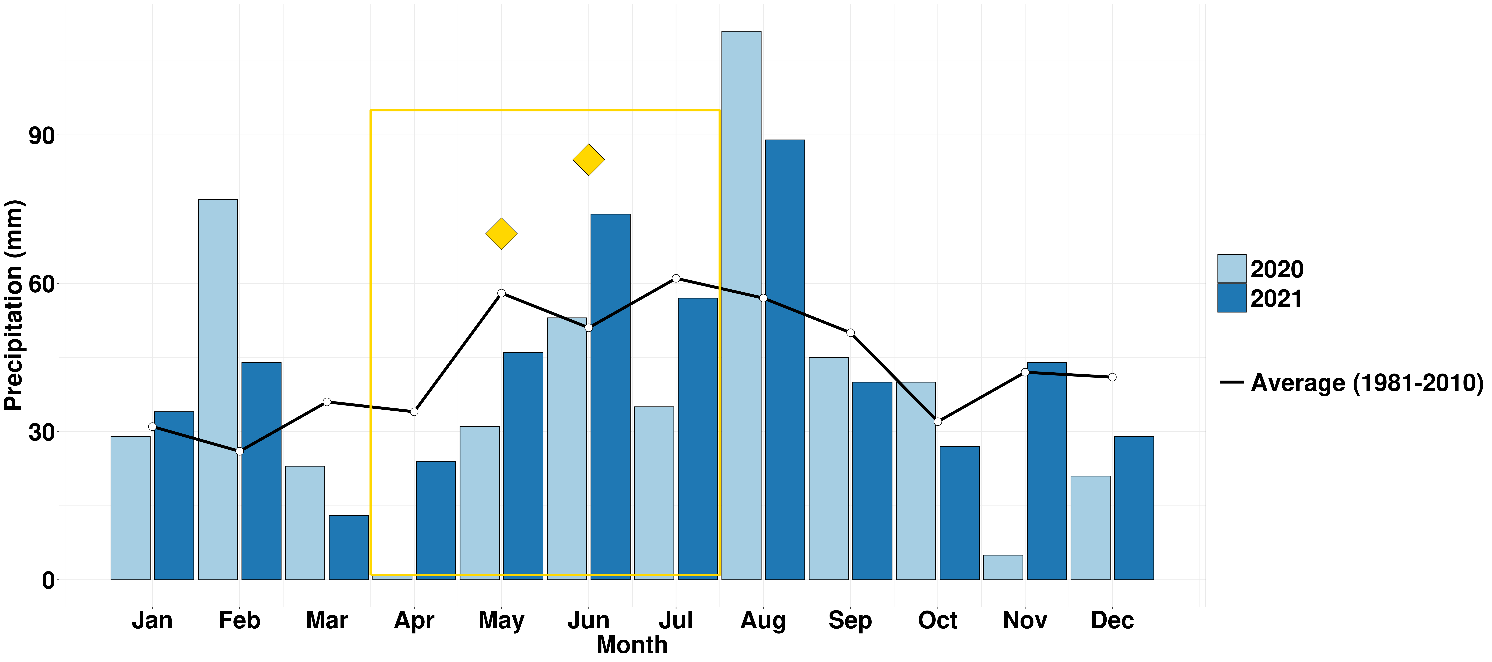


**Figure S4.** The average monthly precipitation at the LTE (long-term experiment) field site in Bernburg, Germany, from 1981 until 2010 and in the experimental years. The yellow box denotes the duration of the field experiments and the diamonds indicate the dates of inoculation with the beneficial microorganism consortium (BMc).


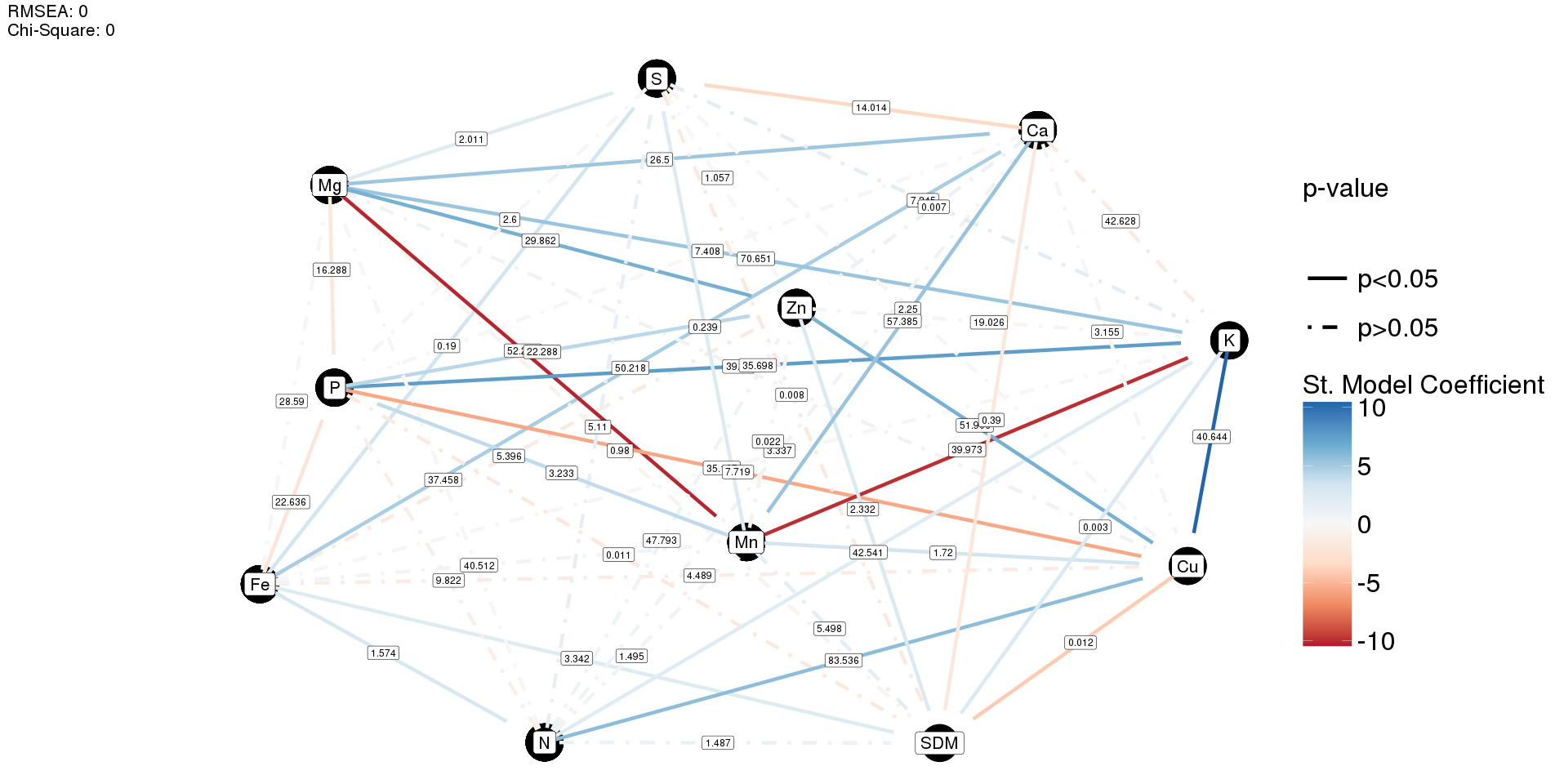


**Figure S5.** Structural equation model of macro- and micronutrient total mass (mg or g kg^-1^ plant), and shoot dry mass (SDM, g plant^-1^) based on partial least squares. The model shows significant relationships with solid lines (*p* ≤ 0.05), dashed lines are not significant. The structural (St.) model coefficient indicates a positive (blue) or negative (red) correlation (n = 64).


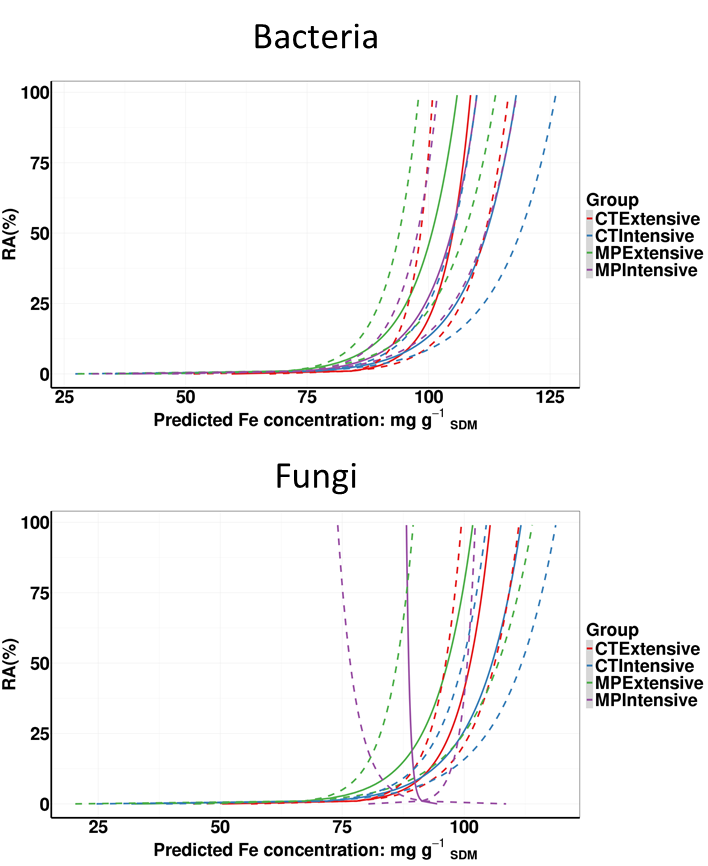


**Figure S6.** Linear model inferring the iron concentration in shoots through the log_10_-transformed relative abundance of iron-associated bacterial (A) and fungal (B) ASVs. The model was examined among the different groups of tillage and N-fertilization intensity to identify model coefficients for each group and error-estimates for each coefficient. Solid lines represent the fit and dashed lines the predicted standard error. For all groups (except MP-Intensive in fungi) similar trends of iron-concentration saturation under high abundances of iron-associated ASVs were detected. SDM: shoot dry mass, MP: mouldboard plough tillage, CT: cultivator tillage, RA: Relative abundance.
